# Spectral Parameters Modulation and Source Localization of Blink-Related Alpha and Low-Beta Oscillations Differentiate Minimally Conscious State from Vegetative State/Unresponsive Wakefulness Syndrome

**DOI:** 10.1371/journal.pone.0093252

**Published:** 2014-03-27

**Authors:** Luca Bonfiglio, Andrea Piarulli, Umberto Olcese, Paolo Andre, Pieranna Arrighi, Antonio Frisoli, Bruno Rossi, Massimo Bergamasco, Maria Chiara Carboncini

**Affiliations:** 1 Department of Translational Research on New Technologies in Medicine and Surgery, School of Physical Medicine and Rehabilitation, University of Pisa, Pisa, Italy; 2 TeCIP Institute, PERCRO Laboratory, Scuola Superiore Sant'Anna, Pisa, Italy; 3 Department of Neuroscience and Brain Technologies, Italian Institute of Technology, Genova, Italy; 4 Department of Medical and Surgical Sciences and Neuroscience, University of Siena, Siena, Italy; Weill Cornell Medical College, United States of America

## Abstract

Recently, the cortical source of blink-related delta oscillations (delta BROs) in resting healthy subjects has been localized in the posterior cingulate cortex/precuneus (PCC/PCu), one of the main core-hubs of the default-mode network. This has been interpreted as the electrophysiological signature of the automatic monitoring of the surrounding environment while subjects are immersed in self-reflecting mental activities. Although delta BROs were directly correlated to the degree of consciousness impairment in patients with disorders of consciousness, they failed to differentiate vegetative state/unresponsive wakefulness syndrome (VS/UWS) from minimally conscious state (MCS). In the present study, we have extended the analysis of BROs to frequency bands other than delta in the attempt to find a biological marker that could support the differential diagnosis between VS/UWS and MCS. Four patients with VS/UWS, 5 patients with MCS, and 12 healthy matched controls (CTRL) underwent standard 19-channels EEG recordings during resting conditions. Three-second-lasting EEG epochs centred on each blink instance were submitted to time-frequency analyses in order to extract the normalized Blink-Related Synchronization/Desynchronization (nBRS/BRD) of three bands of interest (low-alpha, high-alpha and low-beta) in the time-window of 50–550 ms after the blink-peak and to estimate the corresponding cortical sources of electrical activity. VS/UWS nBRS/BRD levels of all three bands were lower than those related to both CTRL and MCS, thus enabling the differential diagnosis between MCS and VS/UWS. Furthermore, MCS showed an intermediate signal intensity on PCC/PCu between CTRL and VS/UWS and a higher signal intensity on the left temporo-parieto-occipital junction and inferior occipito-temporal regions when compared to VS/UWS. This peculiar pattern of activation leads us to hypothesize that resting MCS patients have a bottom-up driven activation of the task positive network and thus are tendentially prone to respond to environmental stimuli, even though in an almost unintentional way.

## Introduction

In the last decade, thanks to functional neuroimaging, neuronal networks involved in consciousness functions have been extensively investigated and characterized both in healthy subjects and in survivors of severe acquired brain injuries with disorders of consciousness (DOC). In the latter, a reduced regional activation in both the extrinsic or task positive network (TPN) (associated with external/sensory awareness and encompassing the dorso-lateral frontoparietal cortices) and the intrinsic or default-mode network (DMN) (associated with internal/self-awareness and encompassing posterior cingulate cortex/precuneus, PCC/PCu, and anterior cingulate cortex/mesiofrontal cortices) has been demonstrated [1], [2], [3], [4], [5], [6]. At the same time, both a reduction of cortico-cortical and thalamo-cortical connectivities within the DMN [7], [8], [9] and of cross-modal interactions between DMN and TPN have also been shown [10]. In particular, the transition from vegetative state/unresponsive wakefulness syndrome (VS/UWS) to minimally conscious state (MCS) (in which behavioural responses to environmental stimuli are consistently/reliably observable) is characterized by intermediate levels of activation between normal and VS/UWS subjects, even though differences between groups have not always proved to be statistically significant across the different studies available in literature [2], [11], [12].

Despite the significant progress made in the understanding of functional deficits underlying the genesis of DOC the differential diagnosis between VS/UWS and MCS is still mainly based on behavioural observations. For this reason, the percentage of misdiagnosis, that is the probability that a subject diagnosed as VS/UWS (i.e., not conscious) can actually possess a certain level of awareness of the self or of the environment (i.e., conscious, albeit at minimum level) still remains too high at present (about 40%) [13]. This is mainly due to the difficulty of detecting behavioural signs of consciousness, which often are weak, rare and controversial, in patients with serious or near-total impairment of motor functions (i.e., motionless and non-cooperative patients). Recently, methods for the detection of covert awareness in such patients have been proposed: fMRI [14], [15], [16] or EEG [17], [18] were used to detect any residual ability to command-following in active paradigms of mental imagery. Although these paradigms have been proven suitable to detect otherwise unrecognized cases of MCS and may pave the way to the development of a basic communication with these patients [13], it cannot be excluded that even individuals unable to organize a detectable cognitive response could display a certain level of self-awareness.

Thus, at present, the perspective of observing a grey area between MCS and VS/UWS (a certain number of false negatives and false positives) still seems a somehow unavoidable bias, virtually inherent to the majority of the proposed markers [8], [19]. In other words, the possibility that a subject with a minimum level of consciousness may elude the correct diagnosis still remains. The current challenge, therefore, is to identify objective and reliable biomarkers able to reduce the rate of misdiagnosis.

Even blink-related delta oscillations (delta BROs), which are originated in the PCC/PCu, as we have shown in a recent paper [20], do not escape this fate: although they were directly correlated to the degree of consciousness impairment and significantly reduced in patients with DOC with respect to normals, they did not prove able to reliably differentiate the MCS subgroup from the VS/UWS subgroup.

On the other hand, it is well established that the human brain responds to events according to a multidimensional pattern, i.e., in both the spatiotemporal and the frequency dimension. Different neuronal populations, either coalescent or spatially distributed, operate their response, either simultaneously or in different time windows, on different frequency bands [21], [22], so that the overall response to the event emerges as the result of the interplay between multiple oscillators at different frequencies. In this framework, we have recently shown that in normal subjects the occurrence of a blink is followed by oscillatory activities in delta and alpha bands with reciprocal and perfectly complementary dynamics [23].

Herein, based on a different statistical approach with respect to that of Bonfiglio and collegues [20], but maintaining the same sample of subjects, we confirm results on delta BROs but we demonstrate that, extending the analysis of blink-related oscillations to frequency bands other than delta (alpha-beta), a reliable discrimination among MCS and VS/UWS subgroups can be obtained. Moreover we identify the cortical sources of alpha-beta activity and we show differences in source localization between healthy subjects, MCS and VS/UWS patients. We discuss these findings on the basis of the functional interpretation of the blinking phenomenon from the point of view of the so-called ‘basic awareness’ [20], [23], [24].

## Materials and Methods

### 1. Ethics statement

The study was approved by the local ethics committee (Comitato Etico Sperimentazione del Farmaco, Azienda Ospedaliero-Universitaria Pisana) and all study protocols were in accordance with the Declaration of Helsinki. Written informed consent for healthy volunteers and patients was obtained from all subjects and legal representatives, respectively.

### 2. Participants

Twelve healthy volunteers (five females), with a mean age of 32.6±13.75 yrs (range 21–63), and nine patients with DOC (five females), with a mean age of 42.9±18.6 yrs (range 21–66), participated in the study. The groups did not differ either by age (*t*-test, *p* = 0.160) or by sex (Fisher Exact Test, *p* = 0.67). To obtain a behavioural differential diagnosis between VS/UWS and MCS, patients received two clinical scales: the JFK Coma Recovery Scale-Revised (CRS-R) [25] and the Level of Cognitive Functioning Scale (LCFS) [26]. The VS/UWS group was composed of 3 subjects with CRS-R score ≤6/23, plus 1 subject with a CRS-R score of 9/23 classified as VS/UWS according to Bruno and collegues [27]. The MCS group consisted of 2 subjects with CRS-R score ≥10/23, plus 3 subjects in whom a CRS-R total score could not be attributed for their LCFS score >4 (see [25]). However, due to their CRS-R partial scores <6 in the Motor subscale and <2 in the Communication subscale, they could be classified as MCS + (see [25]). A synoptic view of patients' characteristics is reported in Table 1. All patients underwent morphologic MRI examination: detected lesions ranged from single or multi-focal lesions (with lobar, sublobar or lacunar extension) to diffuse axonal damage and atrophy, but no focal lesions were detected in correspondence of either the precuneal or the cuneal cortical regions (details about both neuropathology and location of lesions are also reported in Table 1).

**Table 1:** Characteristics of the patients.

Patient	Gender	Age	Diagnosis on admission	Cause of the disorder	Interval since insult (mo)	CSR-R	LCFS	Neuropathology	Lesion Location
**1**	M	64	VS/UWS	ABI	10	5/23	2	A	cortical/subcortical diffuse;
								HD	tri-ventricular;
**2**	F	66	VS/UWS	CVA	96	5/23	2	IBD	b-frontal lobe (subcortical); b-frontal lobe (periventricular);
**3**	M	45	VS/UWS	ABI	51	6/23	2	A	cortical/subcortical diffuse;
								HD	tri-ventricular;
**4**	F	58	VS/UWS	CVA	85	9/23	3	IBD	b-centrum semiovale; b-internal capsule; b-thalamus (postero-medial); b-basal ganglia (caudatum); midbrain (central);
**5**	M	55	MCS	TBI	10	10/23	3	CC/ICH	b-frontal lobe (basal); r-frontal lobe (dorso-lateral posterior); i-temporal lobe; b-basal ganglia;
								DAI	i-frontal lobe (insular); r-temporal lobe (mesial); corpus callosum (splenium); r-thalamus; i-internal capsule; i-midbrain (cerebral peduncule); i-pons (anterior);
								A	cortical/subcortical diffuse;
**6**	F	21	MCS	TBI	21	11/23	3	CC/ICH	-temporal lobe (anterior); r-hypothalamus (paramammilary); r-midbrain (tectum); r-pons (superior/medium cerebellar peduncules);i-temporal lobe (sylvian);
								DAI	i-temporal lobe (posterior); r-temporal lobe (mesial); corpus callosum (genu); r-frontal lobe;
								SDH	r-frontal lobe;
**7**	M	31	MCS+	ABI	176	na	5	A	cortical/subcortical diffuse;
								HD	tri-ventricular;
**8**	F	22	MCS+	TBI	31	na	6	CC/ICH	b-frontal lobe (basal); r-frontal lobe (dorso-lateral posterior) i-temporal lobe; b-basal ganglia;
								DAI	i-frontal lobe (insular); r-temporal lobe (mesial); corpus callosum (splenium); r-thalamus; i-internal capsule; i-midbrain (cerebral peduncule); i-pons (anterior);
								A	cortical/subcortical diffuse;
**9**	F	24	MCS+	TBI	35	na	6	CC/ICH	b-frontal lobe (parasagittal); r-basal ganglia (putamen/pallidum); r-midbrain (cerebral peduncule);
								DAI	b-frontal lobe; b-temporal lobe (inferior); corpus callosum (splenium); i-thalamus; r-internal capsule; b-basal ganglia (caudatum);
								HG	i-frontal lobe

ABI =  anoxic brain injury, CVA =  cerebrovascular accident, TBI =  traumatic brain injury, A = atrophy, HD =  hydrocephalus, IBD =  ischemic brain damage, CC =  cerebral contusion, ICH =  intracerebral hemorrhage, DAI =  diffuse axonal injury, SDH =  subdural hematoma, HG =  hygroma. Data of case numbers 6 and 8 refer to the same subject considered at different levels of clinical severity.

### 3. Recordings

EEG signals were recorded using a BQ132S EEG amplifier (BrainQuick System, Micromed, Treviso, Italy) and an electrode cap (Electro-Cap International, Inc., Eaton, Ohio 45320 USA) at 19 positions following the 10–20 International System. The reference electrode was placed between Fz and Cz (FCz) and electrode impedances were kept below 5 kΩ. EEG signals were acquired at a sampling rate of 256 Hz and band-pass filtered between 0.5 and 45 Hz. Blinks were monitored by means of electro-oculographic (EOG) recordings. EOG electrodes were arranged diagonally to the horizontal line passing by the outer corners of the eyes. Each recording session consisted of approximately 30 minutes of continuously recorded data.

### 4. Experimental set

During the recording session, the subject was seated on a chair (or a wheelchair for some patients) in a noise-insulated room with a comfortable temperature. Subjects were kept unaware that the purpose of the study was focused on spontaneous blinking. They were only instructed to look ahead, letting their eyes wander without paying attention to anything in particular and were left free to think of whatever they wished [20], [23], [24]. Patients were given the same instructions as healthy subjects, whether or not they were able to understand them. As all recordings were made in an eyes open condition, we continuously monitored the ongoing EOG and performed clinical inspection to ensure maintained vigilance. Signal analyses (from pre-processing to data analyses) and statistics were implemented in Matlab (The Mathworks, Natick, MA), while source analyses were performed on the 19 recorded EEG signals using standardized Low Resolution Electromagnetic Tomography (sLORETA) software [29].

### 5. Blinks detection and evaluation

Blink events were automatically detected on the EOG signal on the basis of a correlation-based technique, in which only one sample blink instance had to be manually selected as a template. Whenever the value of the convolution between the template and the signal exceeded a fixed threshold, a blink instance was detected. The minimum delay between two subsequent instances had to be greater than 3 s, to include only artifact-free data segments in the following analyses [20]. All detections were then visually inspected for acceptance or removal.

**Figure 1:**
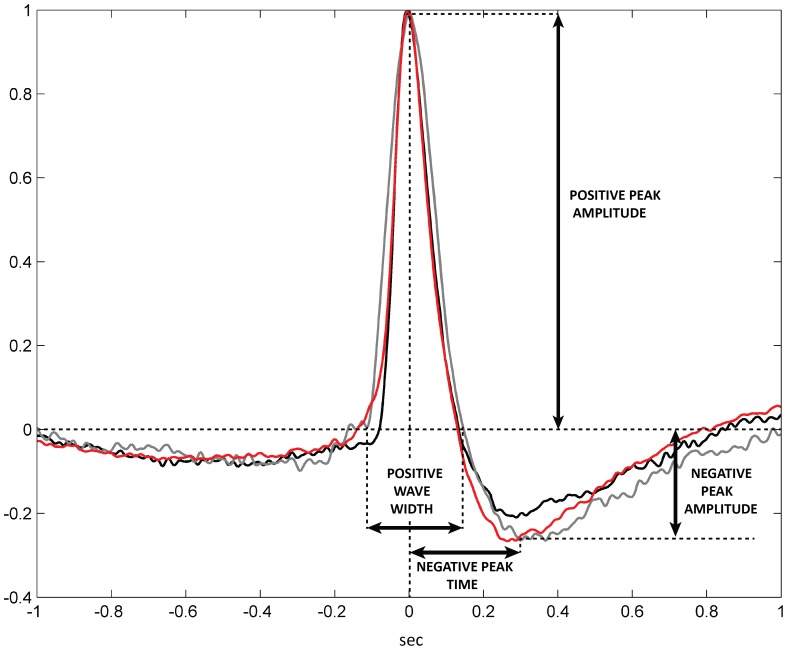
Group-averaged EOG blinks. In the present figure, group-averaged EOG blinks are presented. The red trace refers to CTRL subjects, the gray trace to MCS and the black one to VS/UWS. For each subject the mean blink was normalized to its maximum amplitude. Group-averages were obtained mediating among subjects of the group. In the figure the four features chosen to describe the blink morphology are presented for illustrative purposes (positive peak amplitude, positive wave width, negative peak amplitude, and negative peak time location). Note that statistics on the presented features were perfomed on raw (non-normalized) blinks.

Blinks are characterized by a sharp positive peak followed by a shallow negative deflection. Putative differences in blink shape among the three groups (CTRL, MCS, VS/UWS) were assessed extracting four blink features (see Figure 1 and Table 2): a) positive peak amplitude, b) time distance between the zero-crossing enclosing the positive peak, c) negative deflection minimum amplitude, and d) timing of the negative deflection.

**Table 2:** Descriptive statistics (mean ±2*standard error) and results of ANOVAs with GROUP as a between-subject effect are presented for six parameter describing the EOG blink (significances of F-values are estimated on the basis of permutation tests).

FEATURE	CTRL	MCS	VS/UWS	F-value	p-value
Positive Peak Amplitude (μV)	176±54	187±81	141±25	0.736	0.489
Positive Wave Width (ms)	244±50	272±60	215±41	1.623	0.235
Negative Peak Amplitude (μV)	−25±7	−28.7±11	−23±6	1.293	0.291
Negative Peak Time (ms)	455±11	464±10	466±18	1.501	0.262
Similarity	0.834±0.092	0.833±0.107	0.855±0.045	0.088	0.914
Number of blink trials	90±35	45±18	78±56	1.298	0.296

Each of the extracted features was submitted to a one-way ANOVA with GROUP (CTRL, MCS, VS/UWS) as a between-factor. In order to relax assumptions about data distribution, p-values were estimated on the basis of non-parametric permutation tests [32]: for each of the four bands, 5000 randomly chosen permutations of the original dataset were extracted and their F-statistic was computed. The p-value related to each band was estimated as the ratio between the number of F-values higher than the F-statistics of the original model and the total number of permutations.

Moreover, for each subject, as a measure of the similarity between the selected blinks, the cross-correlation between each couple of blinks was estimated, and the subject mean correlation was extracted. Between-groups differences in similarity were assessed submitting the series of mean cross-correlations to a one-way ANOVA with GROUP as a between-factor analogously to the four morphological features.

Furthermore, we verified if groups did differ in the number of analyzed blinks (i.e., if subjects in the three groups had or not a comparable number of selected blink instances). To this aim the number of blinks per-subject were collected and submitted to an ANOVA with GROUP as a between-factor in the same fashion of the other blink parameters.

### 6. Removal of blink artifacts from the EEG and extraction of blink-related oscillations

**Figure 2:**
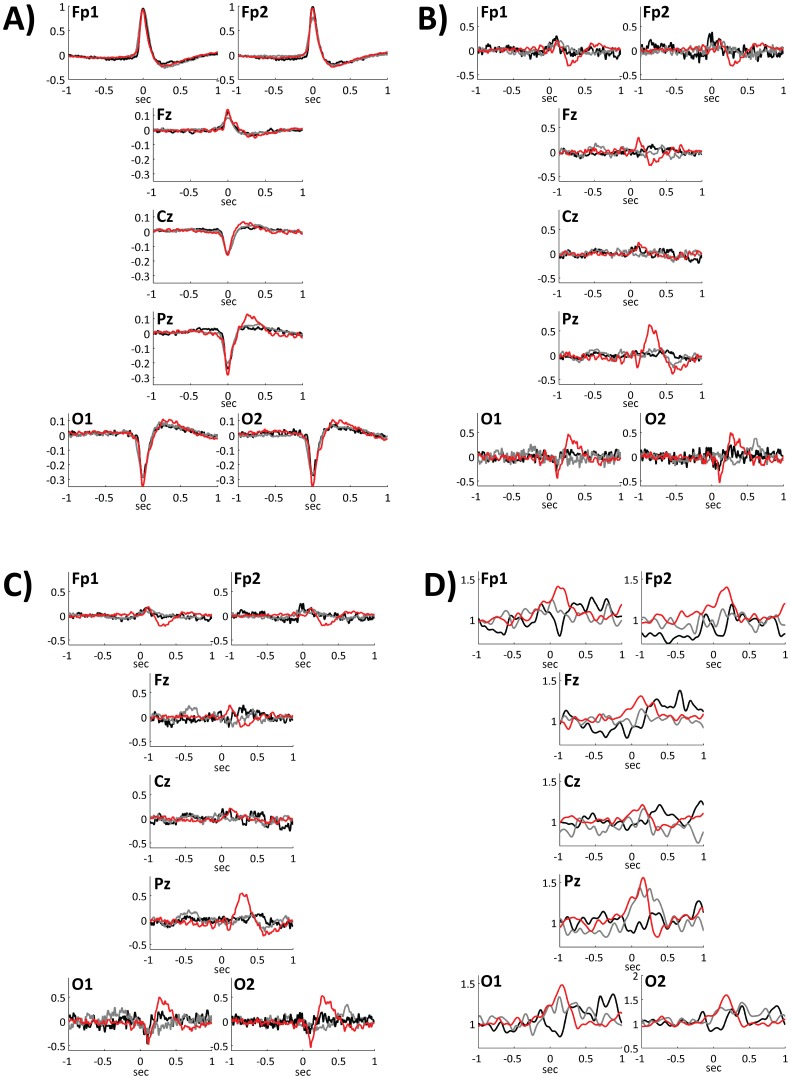
Blink trials: processing steps. *Panel A*: group-averages of raw blink instances (before ICA pruning) are presented for seven electrode sites. Note that for visualization purposes the y-scales of Fp1 and Fp2 graphs are different from those of other electrodes (blink artifacts on those electrodes were obviously much higher than on the other ones). *Panel B*: group-averages of raw blink instances after the blink artifact removal are presented. A BRO with a prominent peak on Pz is already apparent at this stage of processing. *Panel C*: group-averages of blink instances after the blink artifact removal and REST transformation are presented. For all the three panels, the group-averaged signal for each electrode site was obtained mediating between subjects traces. Prior to the group-averaging, traces related to the single subject were normalized to the maximum amplitude of traces over the scalp. *Panel D*: group-averaged ERSPs in low-beta bands are presented for the three groups. In all the four panels red traces indicate CTRL subjects, gray traces MCS subjects and black traces VS/UWS subjects.

For each subject, EEG epochs were extracted in the 3 s interval around the maximum amplitude value (T0) of each EOG blink instance. In Figure 2 (panel A) group averages of the selected blink trials (before blink-artifact removal) are presented for 7 selected electrode sites (Fp1, Fp2, Fz, Cz, Pz, O1 and O2). For each subject and each electrode site, the averaged trial was extracted. Averaged trials from each subject were normalized to the maximum amplitude among the averaged trials. Average trials related to each group were then obtained mediating among subjects of the group. In this and in the following figures, red lines are referred to CTRL group, grey lines to MCS group and black lines to VS/UWS group.

Blink artifacts were removed from non-re-referenced EEG signals by applying an Independent Component Analysis (ICA, runica algorithm [30]), with Principal Component Analysis (PCA) pre-processing, in line with Bonfiglio and colleagues [20]. The effectiveness of the ICA approach in the removal of blink artifacts was demonstrated, in a different framework, by Jung and colleagues [33].

Averaged Blink-Related Oscillations (BROs) [20], [23], [24], obtained after ICA pruning are presented in Figure 2 (panel B). For each subject and each electrode site, the BRO was estimated by averaging across trials. BROs related to each subject were normalized to the maximum BRO amplitude on the scalp. Group-averaged BROs were obtained mediating across subjects. The existence of a BRO peaking on Pz electrode site and detectable only for CTRL subjects is clearly visible in panel B (and, partially, also in panel A).

### 7. EEG referencing

In line with a previous paper from our group [20], surface potentials were referenced off-line (after ICA pruning) to an estimated infinity reference using the REST software [28]. The infinity reference was chosen as this technique has been demonstrated to outperform other commonly used referencing schemes when analyzing power spectra [34], ERP topographies [35], and coherence measures [28].

On the other side, Scalp Current Density transformations (SCD) based on spherical splines have also been proven effective both in avoiding the dependence on the electrical reference choice and in reducing the spatial smearing of the potentials due to the volume conduction of different anatomical structures (i.e., brain, skull, scalp) (see Perrin and colleagues [36]). This approach was demonstrated appropriate also for low density EEG recordings [37], [38].

However, to our best knowledge, a comparison between the performance of REST transformation and SCD has not yet been performed. To render the analyses as robust as possible, and based also on recommendations from Nunez and colleagues [39], we performed the same analyses presented in the manuscript also on SCD-transformed EEG data. Due to space limitations, both the technical details of the chosen SCD transformation and the results of this parallel analysis are presented in the Supporting Information (File S1).

### 8. EEG data analysis

#### 8.1 Blink-related oscillations

For each subject, EEG epochs were analyzed in the time-domain and the corresponding time-locked average potential (BRO) was extracted [20], [23], [24]. In Figure 2 (panel C) group-averaged BROs after the REST transformation are presented. Only for CTRL subjects a prominent peak is apparent on Pz electrode site. In Figure S1 in File S1 (panel C), the analogous blink-related oscillations obtained after SCD transformation are presented.

#### 8.2 Time-frequency analysis

**Figure 3:**
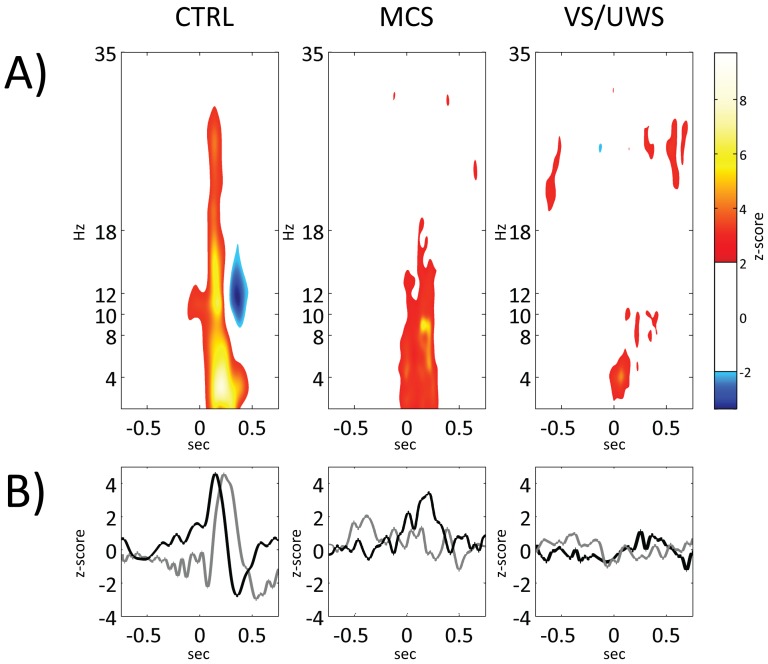
Group-averaged time-frequency z-scores maps (panel A), and time-course of both BRO and low-beta band (panel B). In panel A, groups grand-average z-scores time-frequency maps are depicted; Time-frequency bins with *|z|<1.96*, (*p>0.05*) were left uncoloured. Red to light yellow tones refer to z-scores from 2 to 8, whereas blue tones refer to z-scores less than -2. Each map is obtained as the mean intra-group z-score map (z-scores are evaluated with respect to baseline levels). Each plot of panel B refers to one group (first plot to CTRL, second to MCS and third to VS/UWS). In each plot the time course of both group-averaged *low-beta* z-scores (black line) and of group-averaged BRO (grey line) are depicted. As can be seen, a) the CTRL group shows a significant broadband BRS, followed by a significant BRD only in the 9–17 Hz range; b) the MCS group, shows a significant band-limited (up to 18 Hz) BRS, but totally lacks the subsequent BRD; and, finally, c) the VS/UWS group totally lacks any BRS/BRD.

On the basis of both preliminary observations on BROs (see Figure 2, panels B–C) and of previous findings [20], time-frequency analyses were then focused on Pz electrode. For each subject and each trial, the time-frequency power spectrum was estimated over 1 Hz bins using the Welch method [31]. For each subject, a mean time-frequency power spectrum was obtained by averaging time-frequency power spectra of single trials. Time-frequency bins z-scores were then computed referred to the baseline (1.5 s to 0.5 s before the blink). For each of the three groups (CTRL, MCS and VS/UWS), the grand-average z-score map was estimated (see Figure 3 and Figure S2 in File S1). Based on the examination of time-frequency z-score maps, showing for healthy controls a significant broaband synchronization followed by a band-limited (9-17 Hz) desynchronization, subsequent analyses were conducted for the three bands involved in such a synchronzation-desynchronization sequence: low-alpha (8–10 Hz), high-alpha (10–12 Hz) and low-beta (12–18 Hz). Moreover, to give further evidence about the choice of Pz for time-frequency analyses, Event Related Spectral Perturbations (ERSPs) related to low-beta are presented for seven selected electrode sites (Figure 2, panel D, and Figure S1, panel D, in File S1). ERSPs for each subject and electrode were obtained normalizing the time-frequency signal to its baseline mean value (1.5 to 0.5 s before the blink).

#### 8.3 Normalization of time-frequency synchronization/desynchronization related to the blink

As apparent from healthy subjects (CTRL) group z-score map (Figure 3 panel A, left plot, and Figure S2 in File S1), a significant broadband synchronization happens concurrently with the up-slope of the BRO (from 50 to 300 ms after T0), followed by a band-limited (9–17 Hz) desynchronization during the down-slope (from 300 to 550 ms after T0). For each trial and each band, the difference between log-transformed mean power during the up-slope (50–300 ms after T0) and during the down-slope (300–550 ms after T0) was collected. The difference was normalized to the log-transformed power in the up-down slope interval (50–550 ms after T0); throughout the text we refer to this measure as normalized Blink-Related Synchronization/Desynchronization (nBRS/BRD).Similarly to Bonfiglio and colleagues [20], power in delta band (1–4 Hz) was estimated for each trial in the time-window from 50 to 550 ms and normalized with respect to baseline levels (1.5 to 0.5 s before the blink). Delta series and nBRS/BRD series related to the three other bands of interest, were submitted to weighted least squares one-way ANOVAs with GROUP (CTRL, MCS, VS/UWS) as a three levels between-factor. In these and in the following analyses, the weighting factor for trials related to the 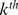 subject was obtained as: 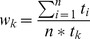, where 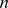 is the total number of subjects and 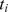 is the number of trials related to the 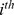 subject. This was done to give an equal statistical weight to subjects within each group, given the high within-group variability in the number of trials [40], [41].

P-values were again estimated on the basis of non-parametric permutation tests [32]: for each of the four bands, 5000 randomly chosen permutations of the original dataset were extracted and their F-statistic was computed. The p-value related to each band was estimated as the ratio between the number of F-values higher than the F-statistics of the original model and the total number of permutations. When appropriate, post-hocs were conducted by applying unpaired t-tests. Also in this case, significance levels were estimated on the basis of non-parametric permutation tests. Sidak correction for multiple comparison [42] was applied to the obtained p-values.

#### 8.4 Source analysis of low-alpha, high-alpha and low-beta bands blink-related activity

Cortical sources of electrical activity in low-alpha, high-alpha and low-beta bands were estimated for each trial in the 500 ms time-window encompassing the BRO peak (50 ms to 550 ms after T0) by means of sLORETA [29], [43], which has been widely used to localize cortical current sources with set-ups consisting of as few as 19 electrodes [20], [44], [45].

For each band, the current density at each voxel was normalized to the voxels current density averaged across all frequencies (0.5–45 Hz) and then log-transformed. Source localization was performed on a three-shell spherical model (Montreal Neurological Institute brain atlas) registered to the Talairach brain atlas [46]. The model consists of 6,239 cortical grey matter voxels at 5 mm resolution. Electrode positions were registered to the spherical model following Towle and colleagues [47]. It's fair to underline that the use of standardized cortical structures instead of the single subject real one (as obtained by MRI) could lead to inaccuracies of source localizations. Valdez-Hernandez and colleagues [48] examined the performances of various standardized head models finding mean localization errors varying between 6 and 9 mm depending on the chosen head model.

On the other side sLoreta has been proven able to reliably identify activations even in deep cortical structures such as cingulate cortices, showing results consistent with PET [49], [50] and fMRI [51] studies.

For each band, differences in current density distributions between the three groups were evaluated computing a weighted one-way ANOVA with GROUP as a 3 levels between-factor for each voxel. Voxels exhibiting a significant GROUP effect were extracted applying a non-parametric single-threshold test (Statistical non-Parametric Mapping, SnPM [52]): the omnibus null hypothesis of no significant between-groups difference in activation levels anywhere in the brain was rejected if at least one F-value (i.e., FMAX) was above the critical threshold FCRIT for p = 0.001 determined by 5000 randomizations (i.e. for each single voxel, 5000 randomly extracted permutations were considered). Voxels in Talairach space with F-values above the critical threshold were considered as yielding significantly different activation levels between groups. For the activated voxels, post-hoc analyses were conducted performing between-groups unpaired t-tests. Critical t-values for a series of t-tests (series being CTRL-MCS, CTRL-VS/UWS and MCS-VS/UWS) were again estimated on the basis of non-parametric single threshold tests with 5000 randomizations and the obtained p-values were adjusted on the basis of Sidak correction [42] for multiple comparisons.

## Results

### 1. EOG blink signals do not differentiate CTRL from MCS and VS/UWS

Both the number of selected blinks per-subject and the intra-subject similarity of selected blinks were not significantly different among the three groups (p>0.29 and p>0.91, respectively) (see Table 2). Four distinctive features of the blink were extracted (for a graphical representation of the selected features see Figure 1) and submitted to ANOVA with GROUP as a between-factor. No significant difference was found for any of the features (features descriptive statistics and ANOVA results are presented in Table 2). Group-averaged blinks are shown in Figure 1. Prior to group-averaging, the averaged EOG blink signal of each subject was normalized to its maximum amplitude for visualization purposes.

### 2. Blink-related oscillations

As apparent from Figure 2 (panels B–C) and Figure S1 in File S1 (panels B–C), a blink-related oscillation is clearly visible for CTRL, but not for either MCS or VS/UWS. The most prominent BRO is found on Pz electrode site regardless of the EEG transformation applied (REST or SCD). This BRO, besides a delta component that was the focus of a previous work [20], is characterized by higher frequency activities as shown both in Figure 2 panel D and Figure S1 panel D in File S1. Both figures depict ERSPs in low-beta band for seven selected electrodes.

### 3. CTRL and MCS groups share a broadband BRS, only the CTRL group shows a band-limited BRD, whereas VS/UWS group shows no BRS/BRD

Group-averaged time-frequency z-score maps referred to the Pz electrode are plotted in Figure 3 (panel A). Time courses of both group-averaged time-frequency z-scores (only low-beta band is depicted for illustrating purposes) and blink-related oscillations z-scores (black and grey lines, respectively) are shown in Figure 3 (panel B). As is apparent from Figure 3 (panel B), only the CTRL group shows a well-defined BRO, which is absent in the other two groups. Furthermore, a) the CTRL group is characterized by a significant broadband (up to 30 Hz) BRS (concurrent with the BRO up-slope), followed by a significant band-limited (9–17 Hz) BRD (concurrent with the BRO down-slope); b) the MCS group, while showing a significant BRS (up to 18 Hz), totally lacks the subsequent BRD; c) the VS/UWS group is characterized by the absence of any BRS/BRD. On the basis of results obtained from the time-frequency evaluation, as already stated in the Materials and methods section, subsequent analyses were focused on those bands that in the CTRL group were involved both in BRS and in BRD: low-alpha (8–10 Hz), high-alpha (10–12 Hz) and low-beta (12–18 Hz). It is worth noting that the same analyses presented in Figure 3, when repeated on SCD-transformed data yield results nearly overlapping those herein presented (see Figure S2 in File S1).

### 4. Delta ERSP discriminates CTRL from MCS and VS/UWS but not MCS from VS/UWS. Low-alpha, high-alpha and low-beta nBRS/BRD levels allow for a complete discrimination between CTRL, MCS and VS/UWS groups

**Figure 4:**
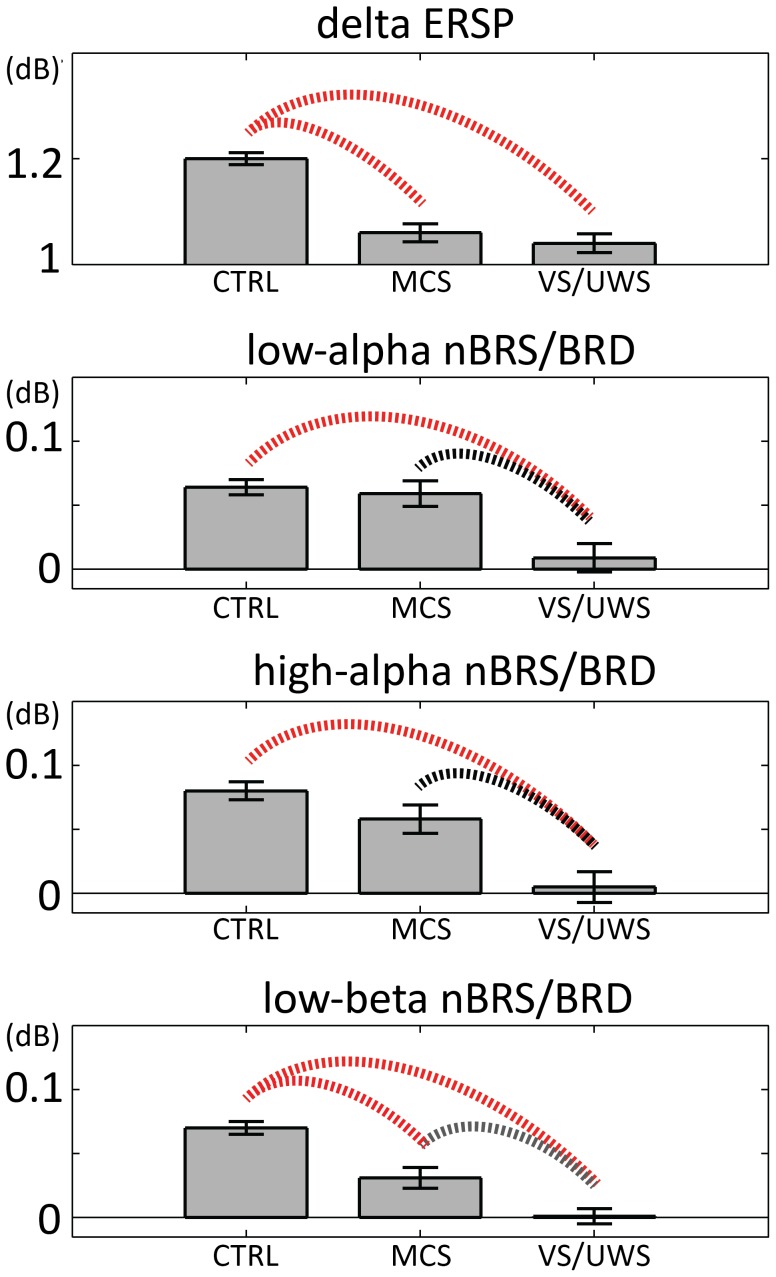
Descriptive statistics of group normalized power and significant between-groups post-hocs. For each band, descriptive statistics (mean ± standard error) of groups normalized power (ERSP for delta and nBRS/BRD for low-alpha, high-alpha and low-beta) and significant between-groups post-hocs are depicted. In each plot the first bar refers to CTRL, the second to MCS and the third to VS/UWS. Only p-values of significant (at least <0.05) post-hocs are highlighted. Red arcs correspond to p<0.001, black arcs to p<0.01 and grey arcs to p<0.05. Regarding delta ERSP, significantly higher levels were found for CTRL both when compared with MCS and VS/UWS. For the other three bands, a significant difference was found both between CTRL and VS/UWS, and between MCS and VS/UWS.

As a first step we verified if delta band allowed for a discrimination between the three groups. To this aim the delta series was submitted to a weighted one-way ANOVA with GROUP as a between factor. A significant group effect was found (p<0.001, see Figure 4), and post-hoc analysis showed that delta power was significantly higher in CTRL than in MCS (p<0.005) and VS/UWS (p<0.001). At variance no difference was found between MCS and VS/UWS, in line with Bonfiglio and colleagues [20]. On the basis of time-frequency analyses, showing for the CTRL group a significant BRS/BRD sequence (synchronized with BRO up and down phases), we verified whether this feature could serve as a marker of differential diagnosis between the groups. To this aim, the nBRS/BRD was estimated for each trial in each of the three bands. For each band, the nBRS/BRD series were submitted to weighted one-way ANOVAs with GROUP (CTRL, MCS, VS/UWS) as a between factor and between-groups post-hoc tests were conducted for significant ANOVAs. As apparent from Figure 4, VS/UWS nBRS/BRD levels are significantly lower than those related to both CTRL and MCS, for all three bands. On the other side, a significant (p<0.001) difference between CTRL and MCS is apparent only for low-beta. The same analysis was conducted on SCD-transformed data and results are presented in File S1. As apparent from Figure S3 in File S1, results about delta ERSP are completely overlapping those presented in the main text, whereas nBRS/BRD of low-alpha, high-alpha and low-beta all significantly differentiate the three groups one from another. As the results obtained from REST-transformed EEG data are more conservative with respect to those obtained from SCD (i.e. the number of significant post-hocs is lower), in the Discussion section we will refer only to the former results.

### 5. Different levels of cortical activation characterize CTRL, MCS and VS/UWS groups

**Figure 5:**
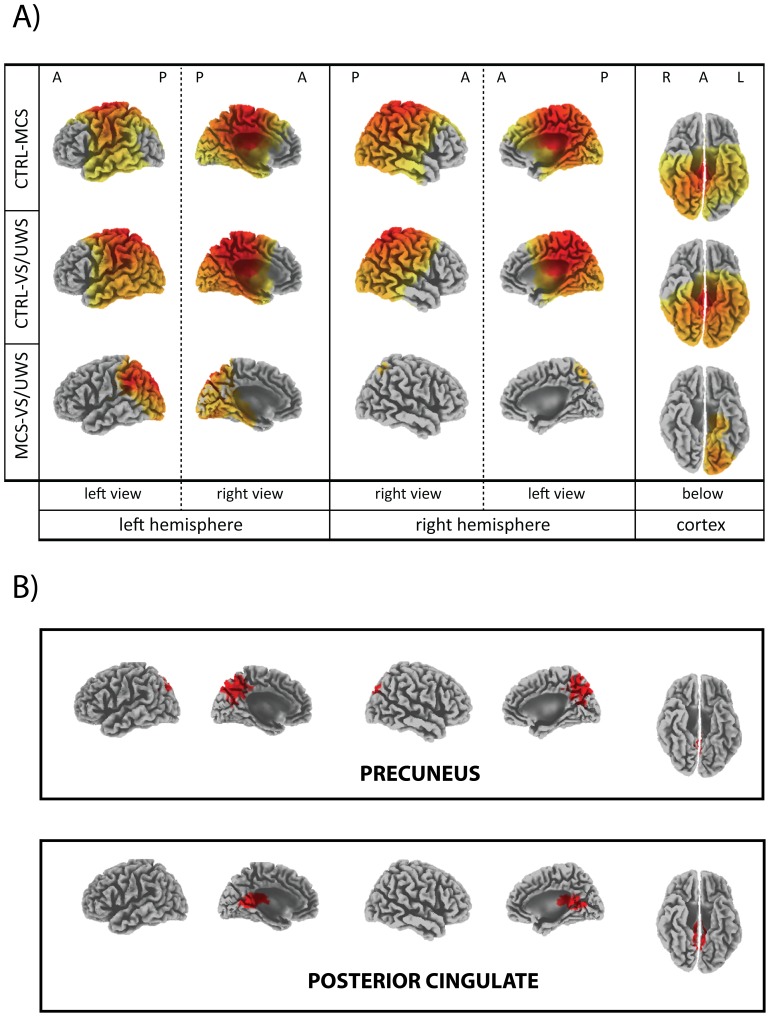
Between-groups post-hocs (CTRL-MCS, CTRL-VS/UWS and MCS-VS/UWS) for low-beta current densities. Results of between-groups post-hocs for low-beta current densities are depicted in panel A. For each group, subject, trial and voxel, the current density value was normalized referred to the full-band current density value of the voxel and then log-transformed. Only voxels with a t-value corresponding to a p-value less than 0.05 are depicted. Yellow to red tones refer to progressively higher t-values: for the CTRL-MCS post-hoc, *4737* voxels had a *p<0.001* on a total of *4774* significant voxels; for the CTRL-VS/UWS post-hoc, *4461* voxels had a *p<0.001* on a total of *4493* significant voxels; for the MCS-VS/UWS post-hoc, *1055* voxels had a *p<0.001* on a total of *1185* significant voxels. Throughout the figure, A denotes the anterior part of the cortex, P the posterior part, R the right hemisphere and L the left one. The CTRL group shows a higher current density on the midline centro-posterior cortices of both hemispheres compared to both MCS and VS/UWS groups. In particular, PCC/PCu (together with anterior cingulate cortex and paracentral lobule) appears as the cortical region where the highest differences are detected in the comparisons between CTRL and DOC, but also between MCS and VS/UWS. Furthermore, CTRL subjects show a higher cortical activity upon the dorsolateral centro-posterior regions of both hemispheres when compared to DOC patients, with the exception of the temporo-parieto-occipital junction (TPOJ) which has current density levels comparable to those of MCS patients. Note that MCS patients show a higher cortical activity on both temporo-parietal junction to VS/UWS. In panel B, the localization of both the precuneus and the posterior cingulate cortex (highlighted in red) are presented as a support to the interpretation of the images presented in panel A.

sLORETA current density estimates at each voxel were collected for each of the three bands of interest and for each trial in a 500 ms time-window encompassing the mean time position of BRO peak (obtained from CTRL subjects). Normalized log-transformed current source densities at each voxel and for each band were submitted to weighted one-way ANOVAs with GROUP (CTRL, MCS, VS/UWS) as a between-factor. Voxels exhibiting a significant GROUP-effect were submitted to post-hoc analyses. Figure 5 (panel A) and Table 3 report results related to low-beta band post-hoc analyses, whereas results related to low-alpha and high-alpha are presented in File S1 (Figure S4 and Table S1, Figure S5 and Table S2 in File S1, respectively). In each figure, the upper panel refers to CTRL-MCS, the central panel to CTRL-VS/UWS and the lower panel to MCS-VS/UWS. In each cortical map, the yellow to red tones refer to progressively higher t-statistics values. Voxels with *|t|<t_crit_* with *t_crit_* corresponding to a *p<0.05* were left uncoloured. As can be seen in Figure 5 (panel A) and Figures S4 and S5 in File S1, (upper and central panels), the CTRL group shows a higher signal intensity upon the midline centro-posterior cortices of both hemispheres when compared to DOC subjects. In particular, PCC/PCu, together with neighboring areas along the midline (i.e., anterior cingulate cortex and paracentral lobule) appears as the cortical region where the most significant differences in the comparisons between CTRL and DOC groups (but also, within this latter group, between MCS and VS/UWS subgroups) are detected. Moreover, CTRL subjects show a higher cortical activity uniformly distributed upon the dorsolateral centro-posterior regions of both hemispheres when compared to VS/UWS patients; when compared to MCS patients, areas with a significantly higher cortical activation are superimposable on those of the previous comparison with the exception of the temporo-parieto-occipital junction (TPOJ), where no significant difference is detected.

**Table 3:** For each of the three groups of post-hocs (CTRL-MCS, CTRL-VS/UWS, MCS-VS/UWS) on low-beta and for each cortical structure, both the number of activated voxels (significantly higher in one group with respect to the other, p<0.05, total number and split per hemisphere and the voxel with the most extreme t-value are presented.

	CTRL-MCS				CTRL-VS/UWS				MCS-VS/UWS			
Cortical structure	Activated voxels			Voxel with higher t	Activated voxels			Voxel with higher t	Activated voxels			Voxel with higher t
	total	LH	RH		total	LH	RH		total	LH	RH	
**Postcentral Gyrus (363)**	363	179	184	3:30,−22,43	363	179	184	3: −20, −27,47	76	74	2	5: −35, −46,58
**Precuneus (355)**	354	160	161	31:20,42,34	355	161	161	7: −5, −32,43	198	160	18	19: −20, −81,41
**Precentral Gyrus (357)**	346	170	176	4:25, −27,47	319	162	157	4: −25, −27,47	2	2	0	4: −30, −27,47
**Superior Temporal Gyrus (419)**	308	179	129	41:35,33,15	278	168	110	41: −35, −33,15	40	40	0	39: −35, −57,30
**Middle Temporal Gyrus (359)**	291	135	156	39:35,62,26	296	173	123	39:35, −62,26	50	50	0	39: −35, −72,27
**Inferior Parietal Lobule (286)**	286	142	144	40:40,32,43	286	142	144	40: −35, −32,38	146	131	15	40: −59, −42,39
**Cingulate Gyrus (287)**	286	114	135	31:20,27,38	263	103	125	31: −15, −27,38	17	17	0	31: −20, −42,25
**Cuneus (273)**	255	106	124	7:10, −66,31	273	124	124	7: −10, −66,31	151	124	2	19: −25, −86,37
**Middle Frontal Gyrus (494)**	247	99	148	6: −15, −7,60	177	79	98	6: −20, −12,60	0	0	0	—
**Fusiform Gyrus (231)**	220	104	116	37:25, −49,10	222	115	107	37: −30, −35, −11	32	32	0	19: −20, −83, −13
**Superior Frontal Gyrus (368)**	197	76	114	6: −15, −11,65	106	48	54	6: −15, −11,65	0	0	0	—
**Medial Frontal Gyrus (354)**	184	63	101	6:10, −22,47	113	49	52	6: −10, −22,47	0	0	0	—
**Parahippocampal Gyrus (185)**	183	93	90	27:15, −34,2	182	93	89	27: −10, −34,2	74	74	0	28: −20, −25, −7
**Insula (209)**	172	92	80	13:30, −33,20	158	90	68	13, −30, −28,20	26	26	0	13: −30, −38,20
**Lingual Gyrus (180)**	158	62	87	19:15, −48,2	180	84	87	19: −15, −44, −2	93	84	0	18: −15, −78,4
**Inferior Temporal Gyrus (158)**	149	74	75	37:45, −68, −1	121	78	43	20: −40, −21, −28	4	4	0	37: −45, −68, −1
**Superior Parietal Lobule (134)**	134	68	66	5:20, −41,57	134	68	66	5: −20, −41,57	73	68	5	7: −25, −75,45
**Posterior Cingulate (87)**	87	39	40	23:5, −28,24	87	39	40	23: −5, −28,24	39	38	0	18: −25, −67,17
**Paracentral Lobule (87)**	87	33	34	31:5, −22,43	87	33	34	5: −15, −36,48	9	9	0	5: −20, −41,48
**Middle Occipital Gyrus (145)**	82	12	70	37:40, −63,3	145	75	70	19: −30, −77,22	69	69	0	19: −30, −77,22
**Inferior Frontal Gyrus (367)**	76	38	38	6:45,2,32	52	28	24	6:45,2,32	0	0	0	—
**Anterior Cingulate (141)**	57	12	31	33:5,11,22	12	3	3	25:0,0, −4	0	0	0	—
**Sub-Gyral (57)**	55	31	24	2:35, −27,38	49	30	19	40:25, −41,53	13	13	0	40: −35, −42,34
**Supramarginal Gyrus (55)**	53	26	27	40:40, −42,34	55	28	27	40: −40, −42,34	28	28	0	40: −45, −42,34
**Uncus (61)**	49	31	18	20: −30, −16,29	47	31	16	20: −30, −16, −29	1	1	0	20: −30, −16,29
**Transverse Temporal Gyrus (36)**	36	18	18	41:40, −33,15	36	18	18	41: −35, −33,11	2	2	0	41: −35, −33,11
**Angular Gyrus (27)**	16	5	11	39:35, −61,35	27	16	11	39: −30, −61,35	16	16	0	39: −30, −61,35
**Inferior Occipital Gyrus (32)**	15	0	15	19:40, −73, −5	32	17	15	18: −25, −88, −12	17	17	0	18: −25, −88, −8
**Subcallosal Gyrus (27)**	15	6	7	34: −25,4, −13	16	9	5	34: −15,4, −13	0	0	0	—
**Superior Occipital Gyrus (14)**	6	0	6	19:35, −81,32	14	8	6	19: −35, −81,32	8	8	0	19: −35, −81,32

Only cortical structures for which at least one of the three groups of post-hocs led to an activation of at least 50% of the total number of voxels are presented. The first column lists the names of cortical structures, whereas the second column lists the total number of voxels related to each cortical structure. For each of the three post-hoc groups, the first column reports the number of activated voxels in each cortical structure, the second column the number of activated voxels in the left hemisphere, the third column the number of activated voxels in the right hemisphere and the fourth column both the Brodmann area of the voxel with the most significant t-value and its Talairach coordinates (X,Y,Z).

On the contrary, as can be observed in Figure 5 and Figures S4 and S5 in File S1 (lower panel), MCS patients show a higher cortical activity only on TPOJ and inferior occipito-temporal regions of the left hemisphere when compared to VS/UWS, with no significant difference on the right hemisphere. Table 3 (and Tables S1–S2 in File S1) reports significant differences in activation levels summarized for each cortical structure. Only cortical structures having a percentage of activated voxels higher than 50% of the total number of voxels pertaining to the structure itself for at least one of the three groups of post-hocs are presented. For each of the three groups of post-hocs (CTRL-MCS, CTRL- VS/UWS, and MCS- VS/UWS) and each cortical structure, both the number of activated voxels and Talairach coordinates and Brodmann area of the voxel with the higher t-value are shown.

## Discussion

### 1. Diagnostic aspects: new blink-related spectral features to assess disorders of consciousness

By inspecting Fig.3, it is readily apparent that the state of full consciousness, corresponding to the healthy condition, is characterized by the possibility of blink-related EEG oscillations ranging from 9 to 17 Hz to be modulated (i.e., synchro- and de-synchronized) by each blink event. This can be considered as a sign of the brain ability to respond to perturbations imposed by the environment (i.e., of its adaptability to the environmental demands). If on one hand that adaptability is almost completely lost in VS/UWS group; on the other hand MCS group is characterized by its partial restoration, as demonstrated by the reappearance of a sychronization (i.e., an increase of the signal power) in the same time and frequency windows of the healthy control subjects (even if not yet followed by a true desyncronization, but only by a simple return to the baseline of the signal intensity).

Moreover, from the examination of Fig.4, low-alpha, high-alpha and low-beta emerge as those frequency bands that enable the differential diagnosis between MCS and VS/UWS, but the frequency bands where such a diagnostic capability is expressed at its highest level are low- and high-alpha. In these bands, however, MCS subjects are statistically indistinguishable from healthy controls, so that it could be argued that in MCS subjects those neuronal assemblies that are capable of producing oscillations in low- and high-alpha bands have already achieved functional levels which are consistent with the condition of normality.

Then, the low-beta band follows, depending on the level of its ability to differential diagnosis. As regards this latter frequency band, however, MCS subjects show significantly lower levels of activation compared with CTRL subjects and, as a consequence, all three subgroups can be differentiated from one another. In other words, in MCS subjects neuronal assemblies which are capable of producing oscillations in this specific frequency band have not yet reached functional levels comparable to healthy control subjects. Assuming that ideally the restoration of full consciousness coincides with the achievement of normal activation levels for all three bands, it could be argued that a) low- and high-alpha oscillations are the first to recover their own modulatory capacity (representing, therefore, a kind of turning point in the transition between unconsciousness and consciousness) and b) by combining the values of alpha and low-beta bands, as if they were two geographical coordinates, it would be theoretically possible to determine the point at which each DOC subject lies along this ideal path at a given time (i.e., to quantify the difference with respect to healthy controls). Thus, identifying and monitoring the different activation patterns progressively achieved by low-alpha, high-alpha and low-beta oscillations could allow us to define with better accuracy the functionality levels regained from time to time by the respective neuronal assemblies along the path of reacquisition of full consciousness, thus providing useful supporting elements to clinical diagnosis.

### 2. Topographic aspects

#### 2.1 Intermediate levels of activation of PCC/PCu characterize MCS subjects

In a previous work, we localized the delta BROs source of normal healthy subjects in PCC/PCu, i.e. at one of the main core-hubs of the default-mode network [20]. This, in the light of the so-called ‘sentinel theory’ [53], has been interpreted as an element in favour of the monitoring function of environmental conditions attributed to the spontaneous blinking at rest [20], [23], [24]. Interestingly, in a recent work Nakano and colleagues [54] showed a transient cortical increase of the BOLD signal in the DMN in relation to the onset of spontaneous blinks of healthy subjects while viewing video stories, thus providing consistent findings with those pertaining to delta BROs [20].

In the present work, the central role of PCC/PCu as the source of blink-related bioelectric brain activity not only is further confirmed, but is also extended to alpha (wide) and beta (low) oscillations. PCC/PCu, together with neighboring areas along the midline (i.e., anterior cingulate cortex and paracentral lobule), in fact, appear as the cortical regions with the most significant differences in activation levels between CTRL and DOC, but also within this latter group, between MCS and VS/UWS, for all the three bands of interest. In particular, MCS subjects show an intermediate level of activation between CTRL and VS/UWS, as though a partial recovery of the functional capabilities that are specific to this region coincided with an improvement of consciousness functions.

### 2.2 MCS subjects also show a preserved activity over left-sided cortical areas

Other cortical regions, however, show a different activation across groups. In particular, healthy subjects show a higher cortical activity than VS/UWS patients, which is symmetrically and uniformly distributed over the centro-posterior regions of the two hemispheres. This distribution is largely the same as that obtained in the comparison with MCS with the exception of the temporo-parieto-occipital junction (TPOJ) of the left hemisphere, where, on the contrary, no significant difference between CTRL and MCS was found. This shows that MCS patients have a reduced cortical activity over the centro-posterior regions of the right hemisphere but not over the TPOJ of the left hemisphere and as such the MCS group is characterized by an interhemispheric functional asymmetry with a relative hyperactivity of the left-sided TPOJ.

Finally, as regards the comparison between MCS and VS/UWS groups, the former shows a higher cortical activity over the temporo-parieto-occipital junction and inferior occipito-temporal regions of the left hemisphere, but not on the centro-posterior regions of the right hemisphere (which means that left-sided TPOJ and inferior occipito-temporal regions are more active than in VS/UWS subjects, but also that the right hemisphere does not statistically differ from VS/UWS subjects and then it is to be understood as functionally depressed in an absolute sense).

Two alternative possibilities can be considered in the explanation of MCS interhemispheric asymmetry: 1) the recovery of cortical activity upon the left hemisphere (temporo-parieto-occipital junction and inferior occipito-temporal regions) is a specific property of MCS subjects; 2) the random aggregation of anatomic lesions among MCS patients may have generated a group biased towards a greater lesion load in the right hemisphere, which, consequently, is relatively less active than the left one. Two orders of factors, however, argue in favor of the first of the two hypotheses, namely: 1) by making a global estimate of the lesions distribution (see Table 1), there does not emerge a clear predominance of the lesion load upon the right hemisphere and 2) our results are entirely consistent with those of Bruno and colleagues [55], who recently showed a preservation of brain metabolism and functional connectivity *in left-sided cortical areas encompassing the language network, premotor, presupplementary motor, and sensorimotor cortices in MCS+ (i.e., patients showing command following) compared to MCS- patients, albeit devoid of clinical verbal or nonverbal expression*. In summary, therefore, the activation of the left temporo-parieto-occipital junction seems a specific prerogative of MCS patients. Furthermore, the higher activation of PCC/PCu in MCS patients when compared to VS/UWS, could be the expression of a greater degree of awareness of the visual-spatial environment, both on a basic and on a global level (Global Gestalt). Conversely, this activation is not comparable to that observed in healthy subjects [56].

We may therefore conclude that: 1) in MCS subjects in resting conditions the mechanism of automatic monitoring of the visuo-spatial environment (‘sentinel’ system) is still deficient, and consequently, the activation of the left TPOJ represents a sort of compensatory mechanism of such a failure, or that 2) the mental activity underlying the resting state of MCS subjects does not require the entry into operation of the sentinel system, simply because such an activity in these subjects is already addressed by itself toward the external environment. The first of the two scenarios would imply a certain intentionality in supporting such a compensatory mechanism, which, however, is not corroborated by our findings; while, as will be discussed later, more than one argument may be advanced in favour of the second possibility.

### 3. Anatomo-functional correlations

#### 3.1 MCS subjects’ attentional resources at rest are attracted by the external environment rather than focused on the internal one

At any time, basic consciousness, meant as the awareness of the self and of the environment, is the result of the functional balance of two anti-correlated systems: the extrinsic network, predominantly activated during the performance of cognitive tasks, and the intrinsic network, mainly activated during non-task-related resting periods [57]. This balance depends on the continuous oscillation of the attentional focus towards either the external or the internal environment and is ruled by a third fronto-parietal system according to current functional requirements [53], [58]. While the subject is immersed in self-reflective thoughts, a minimum level of global attention to the surrounding environment is automatically (i.e., by default) provided by PCC/PCu [53], [59], [60]. Indeed, the ultimate meaning of the entry into operation of this default mechanism is precisely to release a relevant amount of attentional resources to make them available for internal processing. Given these assumptions, the peculiar functional configuration we observed in the MCS group, i.e. a bilateral PCC/PCu underactivation together with a left-sided dorsolateral posterior parietal cortex and inferior occipito-temporal regions hyperactivation, seems to reflect a predominance of the extrinsic network over the intrinsic one. Therefore, contrary to what happens in healthy subjects, where, without perceptual task-oriented demands, attention is spontaneously directed towards the internal environment (i.e., towards self-reflective thoughts), the resting state of MCS subjects seems to be characterized by a prevalent orientation towards the external environment (externally guided cognition). Furthermore, since no sign of functional fronto-parietal connectivity (see Figure S6 in File S1) was detected in these subjects, one might reasonably exclude any sort of top-down control on this type of attentional configuration. As a result, it could be argued that any cognitive processing underlying the activation of these cortical areas in MCS subjects is unconscious/preconscious rather than conscious. It follows that MCS subjects would be substantially devoid of self-referential thinking activities, i.e., that unconstrained flow of thought, proper to the narrative [61] or autobiographical [62] self, that characterizes the normal resting state. It is as though the surrounding environment was capable of capturing (with bottom-up mode) the majority of attentional resources, making them unavailable for introspective thought contents.

Relating all to spontaneous blinking activity, each blink allows either the disengagement of the attentional focus from the previous (visual) perceptive target/object or its engagement to the next one (or, when it takes place during vision of the same stimulus, the renewal of its perception). A behaviour that could be defined exploratory if it was not completely undocked from intentionality, as it is induced by the environment. In any case, such a behaviour enables a differentiated gathering of environmental information, although in all probability not-conscious or pre-conscious. Whatever the meaning of the single blink, two subsequent blinks always delimit an attentional (bottom-up or top-down) temporal span, characterized by a certain level (low or high) of cognitive processing and closed both by the updating and short-term memorization (automatic or executive) of its contents. All this takes on a special meaning if it is admitted, with Crick and Koch [63], that attention and short-term memory represent elementary building blocks of the basic consciousness.

#### 3.2 MCS subjects capture details of visual scenes rather than their Global Gestalt

Moreover, the fact that there is a prevalent hemispheric activation is not incompatible with the gathering of visual information from the surrounding environment as a whole, given that the left hemisphere (fronto-parietal network, posterior parietal cortex, etc.) controls the orientation of visuo-spatial attention to both hemifields (while the right one only to the contralateral hemifield) [64], [65].

The principal characteristic of the left hemisphere, however, is to be predominantly involved in processing local details within complex visual stimuli [66], [67], [68]; that is to say, in the ecological experimental context adopted in our study, the many objects that make up the visual image of the surrounding environment. As a consequence, in these patients, the left hemisphere may play a role in the distinction of the objects (figures) from the background and, therefore, in monitoring the (environmental) relevance of such objects [69], [70]. Recently, Huberle and Karnath [71] have shown that the Global Gestalt of visual scenes is mostly determined by the activation of bilateral temporo-parietal junction, left precuneus and bilateral anterior cingulate cortex. Given that MCS subjects only show an activation of the left temporo-parieto-occipital junction, and an underactivation of PCC/PCu, it could be inferred that they might be able to capture details of visual scenes, but not their whole (i.e., they might be unable to understand the visuospatial context in which they are immersed, while grasping individually the objects that compose it).

#### 3.3 Re-activation of the retroactive memory: object-driven implicit naming and corresponding action recognition

However, the fact that the left TPOJ is functionally coupled to the extrinsic network, gives a prevailing linguistic connotation to the mental activity of MCS subjects, as this cortical region belongs to the language network and is involved in lexical and semantic processes [72], [73]. However, in this phase, as already mentioned, it does not yet seem appropriate to talk about a genuine inner speech linked to the self-narration of consciousness contents. Rather, it is as though MCS patients were required to decrypt the surrounding environment, by recognizing and re-learning it, at the moment of the re-emergence of consciousness functions [71], [74], [75]. On the other hand, human subjects recognize the real world through the experiences and memories (of those experiences) that are stored and appropriately recalled. It is therefore likely that, during the process of recovery of consciousness, the vision of an object evokes first its most fundamental elementary characteristics, as its name (implicit naming) and corresponding action (semantic memory in the strict sense) [76], and, only subsequently, more complex features, such as previous associated experiences (episodic/autobiographical memories evoked by visual cues). Therefore, it should not surprise us that the first step towards the recovery of retroactive memory can pass through a reactivation of semantic memory [55] before episodic/autobiographical memory [77]. In our opinion, this is how the involvement of fusiform and lingual gyri (part of the inferior occipito-temporal associative visual regions), which are included by Mantini and colleagues [78] in the Resting State Network (RSN) 3 (marked by a strong relationship with alpha and beta rhythms and dedicated to visual processing), should be understood.

In our MCS subjects, the activation of these regions coexists with that of parahippocampal regions, which, therefore, in these subjects seem associated with the extrinsic network activity, rather than with the DMN activity as typically happens in the resting state of normal subjects [59], [79]. This would support a possible involvement of the parahippocampal regions in the evocation of semantic memories and mnestic associations by external stimuli. These memories, for the lack of any mechanisms of top-down control, may be suitably defined as unintentional and preconscious memories. These cortical regions are consistent with the recognition (even ‘compulsive’ according to Gerlach and colleagues [80]) and naming of objects [72], [81], [82], [83], the encoding/retrieval of objects associations -among themselves and with (their) context- [84], [85], and the recognition of familiar objects [86]. Functions that can also occur in an automatic/unintentional way for low-demand tasks and memory loads (well) below the capacity limits of working memory [85], [87]. It is interesting to note, in this regard, that the activity in the left temporo-parietal junction has been shown to positively correlate with the inferior occipito-parietal and parahippocampal regions so as to configure a common functional brain network for semantic, autobiographical, and episodic memory retrieval [88].

#### 3.4 Re-activation of the perspective/proactive memory: object-driven planning of simple reaching/grasping movements

Another point that deserves some further attention is the recent demonstration that left fusiform and lingual gyri are also involved in the recognition of manipulable objects [89]. This is a particularly interesting aspect if we consider that MCS subjects are also characterized by the hyperactivation of the left somato-motor resting state network [78], which includes the supplementary motor area and the posterior paracentral lobule (BA 5 m). The latter is known to be involved in the integration of visuo-spatial and proprioceptive information towards reaching movements [90], while supplementary motor area seems to have a preparatory role with respect to relatively simple externally-guided movements in monkeys, such as visually-guided reaching movements [91]. As a consequence, MCS subjects might be engaged in a subcontinuous planning of simple reaching/grasping movements towards neighbouring objects in view of their possible implementation in the immediate future [92], [93], [94]. All this would stress, once again, the MCS subjects' proneness to a potential interactivity with the external environment.

#### 3.5 Towards the reconstruction of a self-narrative thought

Therefore, admitting that MCS subjects have an internal flow of thought, it is likely that this is mainly driven by perceptual information (perceptually guided thought or cognition) rather than internally generated. It is reasonable to assume that the flow of thought does not substantially detach from the surrounding environment, given that, at this stage, only a few fragments of elementary memories (semantic and episodic memories in an embryonic state), directly recalled from the external environment as they are associated with objects that compose it, are available. Possibly, in this phase of recovery, MCS subjects do not yet express that free flow of thought (stimulus-independent-thoughts) which is characteristic of the narrative self, but rather a stream of thought that is still constrained within the limits imposed by sensory perception of environmental objects, without any control from prefrontal top-down signals (thus, to some extent, an unintentional and preconscious thought): a continually recursive and compulsory type of thought, still deeply rooted in and triggered by environmental perceptual aspects (stimulus-dependent-thought).

It could be argued, therefore, that MCS subjects begin their path towards full self-awareness through the reacquisition of the first rudiments of the self-narrative thought (represented by the raw evocation of elementary semantic and/or associative memories, and by a draft of proactive thought), which only later, will be able to free itself from the constraints that bind it to the perceived environment to follow its own self-determined and totally unconstrained (spontaneous) flow, that is generated by the default-mode network activity.

## Conclusions

This leads us to tentatively put forward some speculations. The ultimate consequences of this reasoning would see the subject in MCS as tendentially prone to a response to environmental stimuli (even though in an almost uncritical, compulsory or unintentional way) and therefore able to get relatively higher scores in clinical rating scales (such as the CRS-R), such as to justify their belonging to a superior diagnostic category than VS/UWS, but poorly provided with (self-)awareness. This interpretation seems also consistent with the behavioural characteristics described for levels III-V of the Level of Cognitive Functioning Scale (LCFS) [26], which is usually employed for the clinical assessment of MCS patients, and the results of a recent study by Monti and colleagues [13], which showed that, paradoxically, only one out of 31 MCS subjects (3%) was able to perform a mental imagery task versus 4 out of 23 subjects with a behavioural diagnosis of VS/UWS (17%).

On the other hand, we should admit the theoretical possibility that some subjects with a functional pattern of the brain which was totally biased towards a relative hyperactivation of the default-mode network due to a functional deficit of the extrinsic/task-positive network (subjects in which the alternation between DMN and TPN is not possible either for a TPN failure or for an impairment of the fronto-parietal system that controls the switch) could be totally irresponsive to the environment and therefore subject to a clinical misclassification as VS/UWS, despite being provided with some form of self-awareness. We could speak, in accordance to Bruno and colleagues [95], of a sort of ‘functional’ locked-in syndrome. This would take confirmation from what has been recently described in the literature about a patient behaviourally diagnosed as VS/UWS which surprisingly showed some activity/connectivity in the DMN [5].

Furthermore, such a scenario would require a reevaluation of the delta BROs role in the study of consciousness functions, over and above the fact that this parameter did not previously allow the differentiation of MCS from VS/UWS subjects [20]. Delta BROs would represent, in fact, a sign of the optimum integration between the intrinsic and the extrinsic systems as found in normal conditions at rest, i.e. the function of the automatic (by default) monitoring of the surrounding environment exerted by PCC/PCu during (self)reflective thought activities [20], [23], [24]. This, as we have seen, belongs, to a very small extent, to MCS as well as to VS/UWS subjects.

In the light of what has been discussed, in conclusion, it can be argued that a) the most promising strategy in the evaluation of patients with DOC seems to be at present that of combining together complementary paradigms, related to the function of both the extrinsic network (active and passive paradigms) and the intrinsic network (resting paradigms), so as to minimize the number of false negatives and false positives and achieve the maximum amplification of diagnostic capabilities; and b) in order to investigate either functional or dysfunctional aspects of consciousness in a more exhaustive way by means of the blink-related EEG analysis, the reciprocal dynamics of both medium-high frequency (alpha-beta) and low frequency (delta) oscillations should be taken into account.

## Supporting Information

File S1 **Contains Figures S1, S2, S3, S4, S5 and S2 and Tables S1 and S2. In this file the same analyses shown in the manuscript have been carried out on SCD-transformed EEG signals.**
(DOC)

File S2 **Contains three exemplary EEG recordings in Matlab format, one related to a healthy subject, one to a MCS/UWS patient and one to a VS patient.**
(ZIP)
