# Correction: Spectral Parameters Modulation and Source Localization of Blink-Related Alpha and Low-Beta Oscillations Differentiate Minimally Conscious State from Vegetative State/Unresponsive Wakefulness Syndrome

**DOI:** 10.1371/journal.pone.0095948

**Published:** 2014-04-23

**Authors:** The PLOS ONE Staff

There is an error in the legend for File S2. Please see the correct legend here.

## Supporting Information

File S2 **Contains three exemplary EEG recordings in Matlab format, one related to a healthy subject, one to a MCS patient and one to a VS/UWS patient.**
(ZIP)

## References

[1] Bonfiglio L, Piarulli A, Olcese U, Andre P, Arrighi P, et al (2014) Spectral Parameters Modulation and Source Localization of Blink-Related Alpha and Low-Beta Oscillations Differentiate Minimally Conscious State from Vegetative State/Unresponsive Wakefulness Syndrome. PLoS ONE 9(3): e93252 doi:10.1371/journal.pone.0093252 24676098 10.1371/journal.pone.0093252 PMC3970990

