# Supplementary material for: Spectral Parameters Modulation and Source Localization of Blink-Related Alpha and Low-Beta Oscillations Differentiate Minimally Conscious State from Vegetative State/Unresponsive Wakefulness Syndrome
Source: PLoS One. 2014 Mar 27;9(3):e93252. doi: 10.1371/journal.pone.0093252 (PMC3970990; doi:10.1371/journal.pone.0093252)
Supplement: File S1 — Contains Figures S1, S2, S3, S4, S5 and S2 and Tables S1 and S2. In this file the same analyses shown in the manuscript have been carried out on SCD-transformed EEG signals. (DOC) [file pone.0093252.s001.doc]

**Supporting Information**

**Scalp Current Densities estimation**

EEG scalp potential distributions are strongly affected by the choice of the electrical reference, which becomes even more crucial when dealing with low spatial resolution recordings (small number of electrode sites) as it is often the case in clinical practice. Current source density transformation based on spherical splines (Perrin et al., 1989, 1990), has been proven effective in avoiding the dependence on the electrical reference choice and in reducing the spatial smearing of the potential due to the volume conduction of different anatomical structures (i.e. brain, skull, scalp, see Perrin et al., 1987).. This approach was demonstrated appropriate also for low density EEG recordings (Kayser and Tenke, 2006; Cincotti et al., 2004).

On the basis of these considerations and after the blink artifact removal, scalp we choose to perform the same analyses presented in the manuscript also on current densities-transformed EEG data (SCD). Current densities were estimated from surface EEG potentials using surface spline transformation with previously established computation parameters (4^th^ degree surface spline and smoothing constant λ set to 10^-5^, CSD Toolbox, Kayser and Tenke, 2006).

Analyses presented in Figures A, B, C are performed on SCD transformed data.

# References

1. Perrin F, Pernier J, Bertrand O, Echallier JF (1989) Spherical splines for scalp potential and current density mapping. Electroencephalogr Clin Neurophysiol 72(2): 184-7

2. Perrin F, Pernier J, Bertrand O, Echallier JF (1990) Corrigendum EEG 02274. Electroencephalogr Clin Neurophysiol 76: 565

3. Perrin F, Bertrand O, Pernier J (1987) Scalp Current Density Mapping: Value and Estimation from Potential Data. IEEE Trans Biomed Eng 34(4): 283-7

4. Kayser J, Tenke CE (2006) Principal components analysis of Laplacian waveforms as a generic method for identifying ERP generator patterns: II. Adequacy of low-density estimates. Clinical Neurophysiology 117: 369-80

5. Cincotti F, Babiloni C, Miniussi C, Carducci F, Moretti D, Salinari S, Pascual-Marqui R, Rossini PM, Babiloni F (2004) EEG deblurring techniques in a clinical context. Methods Inf Med, 43(1):114-7


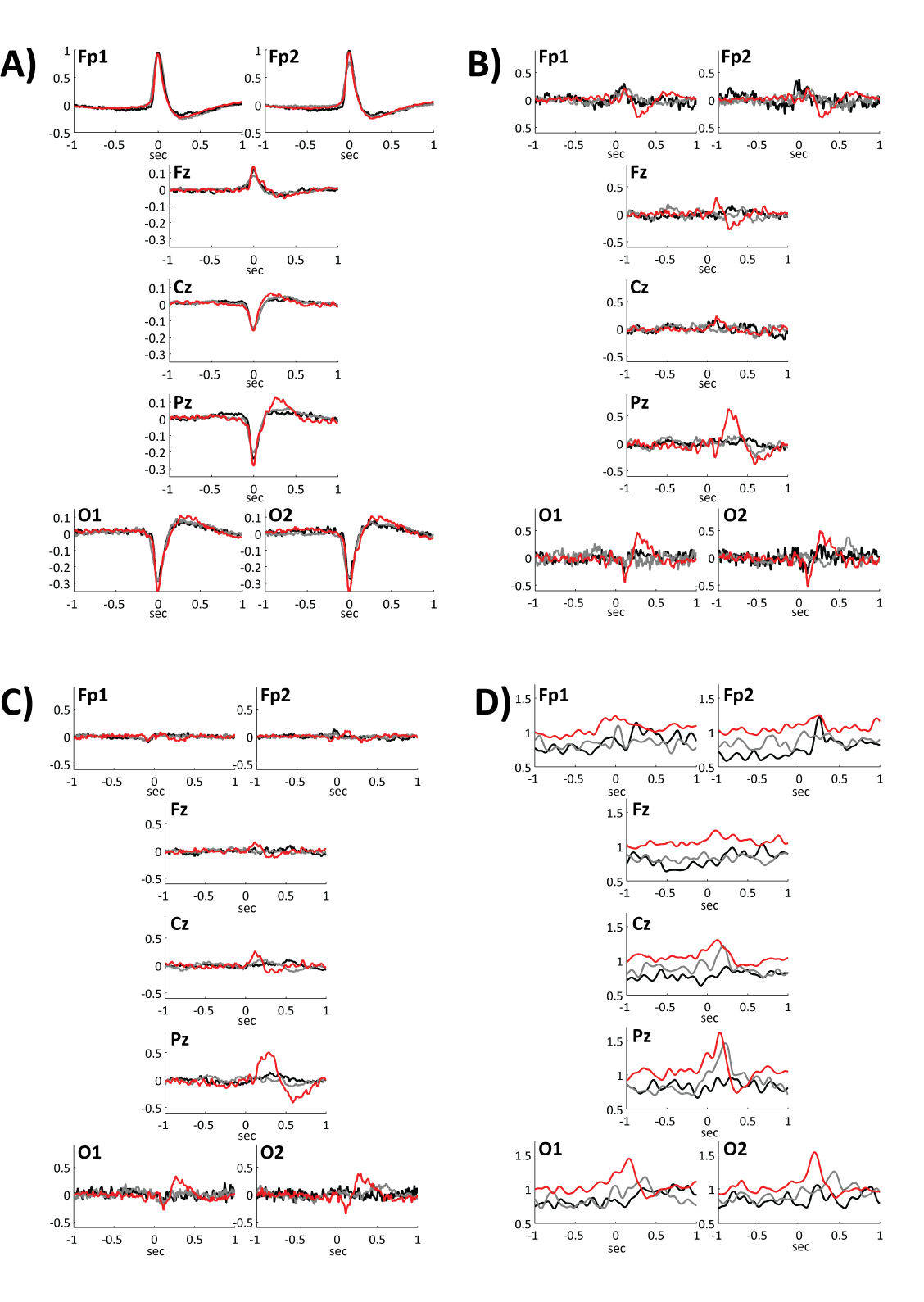


**Figure S1: Blink trials: processing steps. [corresponding to Figure 2 of the MS].**

*Panel A*: group-averages of raw blink instances (before ICA pruning) are presented for seven electrode sites. Note that for visualization purposes the y-scales of Fp1 and Fp2 graphs are different from those of other electrodes (blink artifacts on those electrodes were obviously much higher than on the other ones).

*Panel B:* group-averages of raw blink instances after the blink artifact removal are presented. A BRO with a prominent peak on Pz is already apparent at this stage of processing.

*Panel C:* group-averages of blink instances after the blink artifact removal and SCD transformation are presented.

For all the three panels, the group-averaged signal for each electrode site was obtained mediating between subjects traces. Prior to the group-averaging, traces related to the single subject were normalized to the maximum amplitude of traces over the scalp.

*Panel D:* group-averaged ERSPs in low-beta bands are presented for the three groups.

In all the four panels red traces indicate CTRL subjects, gray traces MCS subjects and black traces VS/UWS subjects.


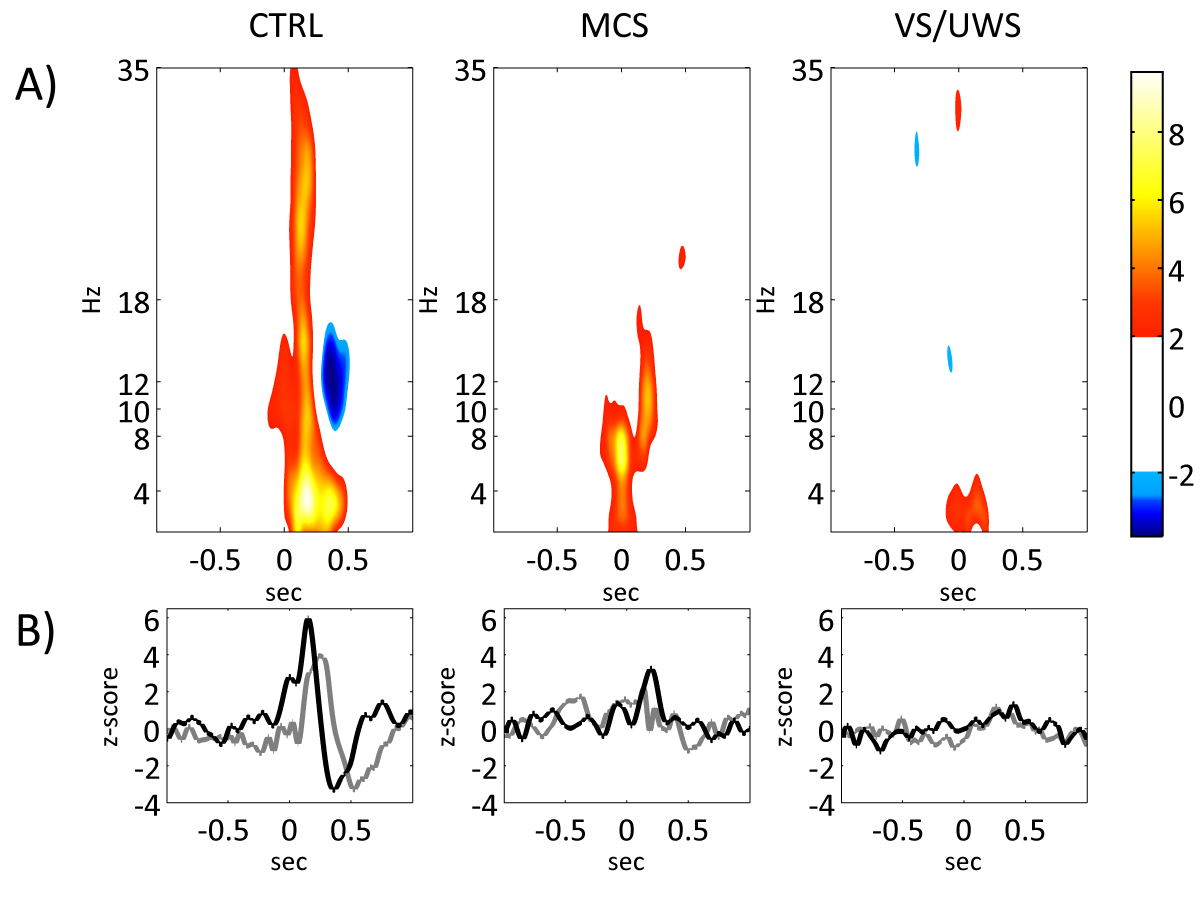


**Figure S2: Group-averaged time-frequency z-scores maps (panel A), and time-course of both BRO and low-beta band (panel B). [corresponding to Figure 3 of the MS]**

In panel A, groups grand-average z-scores time-frequency maps are depicted; Time-frequency bins with *|z| < 1.96*, (*p > 0.05)* were left uncoloured. Red to light yellow tones refer to z-scores from 2 to 8, whereas blue tones refer to z-scores less than -2. Each map is obtained as the mean intra-group z-score map (z-scores are evaluated with respect to baseline levels). Each plot of panel B refers to one group (first plot to CTRL, second to MCS and third to VS/UWS). In each plot the time course of both group-averaged *low-beta* z-scores (black line) and of group-averaged BRO (grey line) are depicted.

As can be seen, a) the CTRL group shows a significant broadband BRS, followed by a significant BRD only in the 9-17Hz range; b) the MCS group, shows a significant band-limited (up to 18Hz) BRS, but totally lacks the subsequent BRD; and, finally, c) the VS/UWS group totally lacks any BRS/BRD.


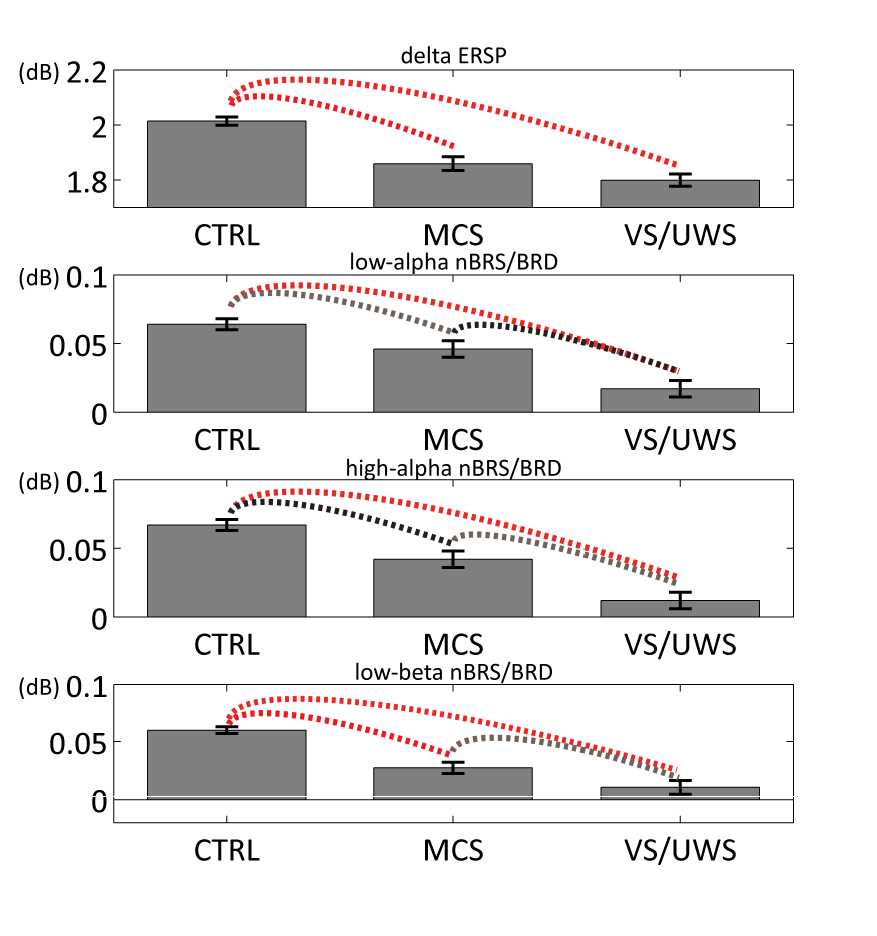


**Figure S3:** **Descriptive statistics of group normalized power and significant between-groups post-hocs. [corresponding to Figure 4 of the MS]**

For each band, descriptive statistics (mean ± 2*standard error) of groups normalized power (ERSP for delta and nBRS/BRD for low-alpha, high-alpha and low-beta) and significant between-groups post-hocs are depicted. In each plot the first bar refers to CTRL, the second to MCS and the third to VS/UWS. Only p-values of significant (at least < 0.05) post-hocs are highlighted. Red arcs correspond to p < 0.001, black arcs to p < 0.01 and grey arcs to p < 0.05.

Regarding delta ERSP, significantly higher levels were found for CTRL both when compared with MCS and VS/UWS. For the other three bands, a significant difference was found between CTRL and VS/UWS, between CTRL and MCS and between MCS and VS/UWS.


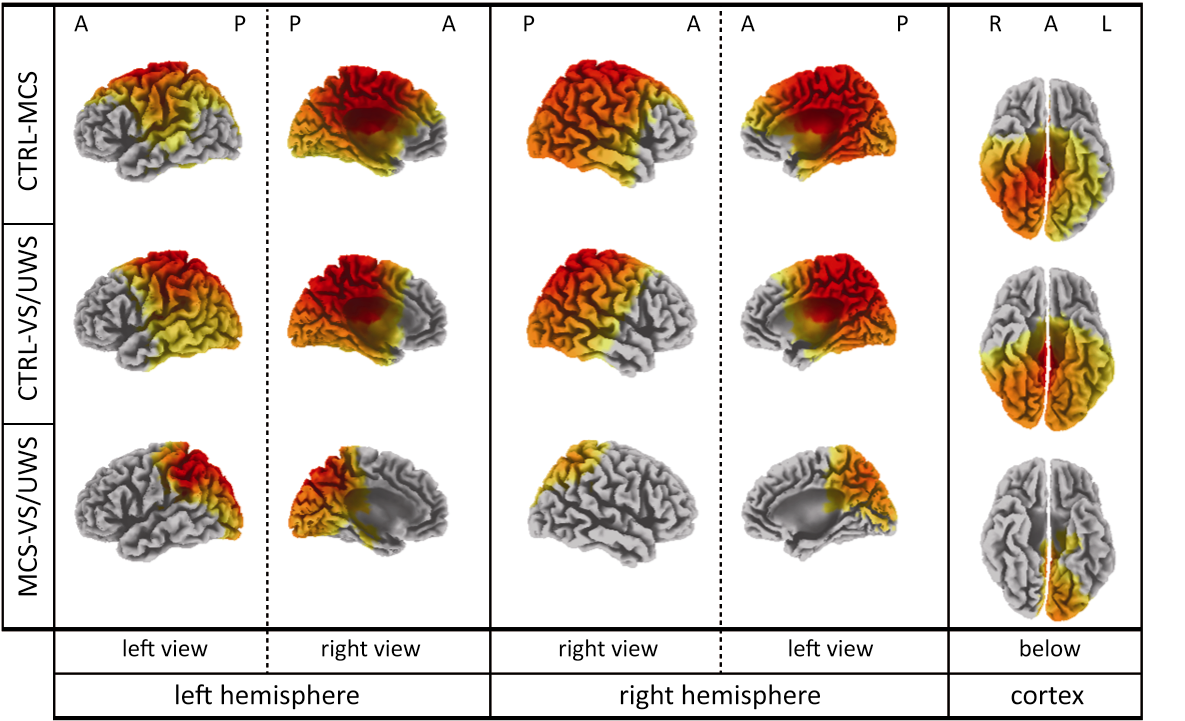


**Figure S4:** Results of between-groups post-hocs for low-alpha current densities are depicted. For each group, subject, trial and voxel, the current density value was normalized referred to the full-band current density value of the voxel and then log-transformed. Only voxels with a t-value corresponding to a p-value less than 0.05 are depicted. Yellow to red tones refer to progressively higher t-values: for the CTRL-MCS post-hoc, 4670 voxels had a p < 0.001 on a total of 4734 significant voxels; for the CTRL-VS/UWS post-hoc, 4322 voxels had a p < 0.001 on a total of 4334 significant voxels; for the MCS-VS/UWS post-hoc, 1885 voxels had a p < 0.001 on a total of 1999 significant voxels. Throughout the figure, A denotes the anterior part of the cortex, P the posterior part, R the right hemisphere and L the left one.


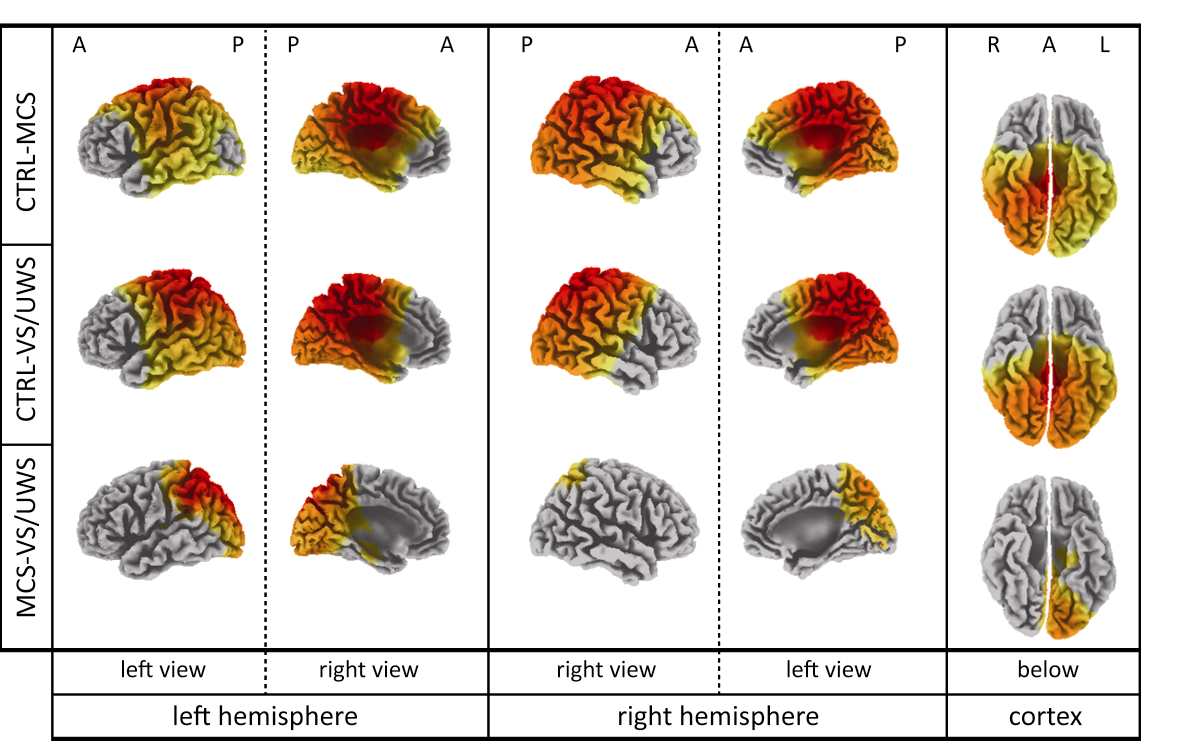


**Figure S5:** Results of between-groups (CTRL, MCS and VS/UWS) post-hocs for high-alpha current densities are depicted. For each group, subject, trial and voxel, the current density value was normalized referred to the full-band current density value of the voxel and then log-transformed. Upper panel refers to CTRL-MCS post-hocs, central panel to CTRL-VS/UWS and lower panel to MCS-VS/UWS. Only voxels with a t-value corresponding to a p-value less than 0.05 are depicted. Yellow to red tones refer to progressively higher t-values: for the CTRL-MCS post-hoc, 4990 voxels had a p < 0.001 on a total of 5028 significant voxels; for the CTRL-VS/UWS post-hoc, 4408 voxels had a p < 0.001 on a total of 4429 significant voxels; for the MCS-VS/UWS post-hoc, 1421 voxels had a p < 0.001 on a total of 1586 significant voxels. Throughout the figure, A denotes the anterior part of the cortex, P the posterior part, R the right hemisphere and L the left one. First columns show a left view of the left hemisphere, second columns a right view of the left hemisphere, third columns a right view of the right hemisphere, fourth columns a left view of the right hemisphere and fifth columns a view from below of the whole cortex.

**Table S1**

|  | **CTRL-MCS** | | | | **CTRL-VS/UWS** | | | | **MCS-VS/UWS** | | | |
| --- | --- | --- | --- | --- | --- | --- | --- | --- | --- | --- | --- | --- |
| **Cortical**  **structure** | **activated voxels** | | | **voxel with**  **higher t** | **activated voxels** | | | **voxel with higher t** | **activated voxels** | | | **voxel with**  **higher t** |
|  | **total** | **LH** | **RH** |  | **total** | **LH** | **RH** |  | **total** | **LH** | **RH** |  |
| **Postcentral Gyrus (363)** | 363 | 179 | 184 | 3:20,-27,52 | 363 | 179 | 184 | 3:20,-31,52 | 239 | 145 | 94 | 5:-35,-46,58 |
| **Precuneus (355)** | 355 | 161 | 161 | 7:5,-32,43 | 355 | 161 | 161 | 7:15,-41,48 | 351 | 161 | 157 | 19:-20,-81,36 |
| **Precentral Gyrus (357)** | 338 | 161 | 177 | 4:20,-22,52 | 300 | 148 | 152 | 4:25,-27,47 | 74 | 55 | 19 | 4:-30,-27,47 |
| **Cingulate Gyrus (287)** | 287 | 115 | 135 | 24:15,-18,38 | 273 | 109 | 128 | 31:15,-32,43 | 108 | 46 | 44 | 31:-20,-32,38 |
| **Inferior Parietal Lobule (286)** | 286 | 142 | 144 | 40:35,-41,53 | 286 | 142 | 144 | 40:35,-41,53 | 233 | 133 | 100 | 40:-35,-41,48 |
| **Middle Frontal Gyrus (494)** | 282 | 127 | 155 | 6:-15,-7,60 | 173 | 83 | 90 | 6:-20,-12,60 | 0 | 0 | 0 | -- |
| **Cuneus (273)** | 272 | 123 | 124 | 7:10,-66,31 | 273 | 124 | 124 | 7:10,-66,31 | 248 | 124 | 99 | 19:-25,-86,37 |
| **Superior Temporal Gyrus (419)** | 244 | 99 | 145 | 41:35,-33,15 | 223 | 116 | 107 | 39:35,-57,30 | 35 | 31 | 4 | 39:-35,-57,30 |
| **Medial Frontal Gyrus (354)** | 231 | 91 | 116 | 6:15,-12,47 | 115 | 50 | 52 | 6:10,-27,52 | 18 | 7 | 8 | 6:-10,-27,52 |
| **Superior Frontal Gyrus (368)** | 224 | 95 | 122 | 6:-15,-11,65 | 117 | 55 | 58 | 6:-20,-11,65 | 0 | 0 | 0 | -- |
| **Fusiform Gyrus (231)** | 220 | 104 | 116 | 37:30,-49,10 | 219 | 111 | 108 | 19:25,-54,-10 | 20 | 20 | 0 | 18:-20,-88,-12 |
| **Middle Temporal Gyrus (359)** | 211 | 42 | 169 | 39:35,-62,26 | 259 | 132 | 127 | 39:35,-62,26 | 40 | 40 | 0 | 39:-35,-72,27 |
| **Parahippocampal Gyrus (185)** | 185 | 93 | 92 | 27:15,-34,2 | 181 | 93 | 88 | 19:20,-48,2 | 43 | 43 | 0 | 28:-25,-20,-7 |
| **Lingual Gyrus (180)** | 177 | 81 | 87 | 18:20,-53,7 | 180 | 84 | 87 | 18:20,-53,7 | 113 | 83 | 21 | 17:-10,-87,4 |
| **Insula (209)** | 171 | 81 | 90 | 13:30,-28,20 | 142 | 79 | 63 | 13:-30,-28,20 | 28 | 28 | 0 | 13:-30,-28,20 |
| **Superior Parietal Lobule (134)** | 134 | 68 | 66 | 5:20,-41,57 | 134 | 68 | 66 | 19:30,-77,22 | 134 | 68 | 66 | 7:-30,-46,58 |
| **Inferior Temporal Gyrus (158)** | 102 | 22 | 80 | 19:50,-58,-1 | 108 | 64 | 44 | 37:45,-68,-1 | 0 | 0 | 0 | -- |
| **Posterior Cingulate (87)** | 87 | 39 | 40 | 23:5,-28,24 | 87 | 39 | 40 | 31:5,-52,30 | 68 | 39 | 21 | 31:-10,-67,17 |
| **Paracentral Lobule (87)** | 87 | 33 | 34 | 31:10,-12,47 | 87 | 33 | 34 | 5:15,-36,48 | 77 | 30 | 31 | 5:-20,-41,48 |
| **Middle Occipital Gyrus (145)** | 81 | 11 | 70 | 37:40,-63,3 | 145 | 75 | 70 | 5:20,-41,57 | 67 | 61 | 6 | 18:-15,-87,18 |
| **Anterior Cingulate (141)** | 74 | 23 | 35 | 33:5,11,22 | 20 | 5 | 7 | 33:-5,11,22 | 0 | 0 | 0 | -- |
| **Inferior Frontal Gyrus** | 59 | 17 | 42 | 6:45,2,32 | 26 | 10 | 16 | 6:45,2,32 | 0 | 0 | 0 | -- |
| **Uncus (61)** | 56 | 29 | 27 | 20:30,-16,-24 | 38 | 29 | 9 | 34:-15,-6,-21 | 0 | 0 | 0 | -- |
| **Sub-Gyral (57)** | 56 | 32 | 24 | 6:20,-7,56 | 49 | 30 | 19 | 40:25,-41,53 | 21 | 12 | 9 | 7:-25,-46,53 |
| **Supramarginal Gyrus (55)** | 51 | 24 | 27 | 40:40,-42,34 | 55 | 28 | 27 | 40:40,-47,35 | 32 | 28 | 4 | 40:-40,-42,34 |
| **Transverse Temporal Gyrus (36)** | 36 | 18 | 18 | 41:40,-33,15 | 36 | 18 | 18 | 41:40,-33,15 | 3 | 3 | 0 | 41:-35,-33,11 |
| **Angular Gyrus (27)** | 20 | 9 | 11 | 39:35,-61,35 | 27 | 16 | 11 | 19:40,-73,-5 | 21 | 16 | 5 | 39:-30,-61,35 |
| **Subcallosal Gyrus (27)** | 17 | 8 | 7 | 25:0,4,-13 | 14 | 8 | 4 | 39:35,-61,35 | 0 | 0 | 0 | -- |
| **Inferior Occipital Gyrus (32)** | 16 | 1 | 15 | 19:40,-73,-5 | 32 | 17 | 15 | 34:-10,4,-13 | 17 | 17 | 0 | 17:-20,-93,-8 |
| **Superior Occipital Gyrus (14)** | 8 | 2 | 6 | 19:35,-81,32 | 14 | 8 | 6 | 19:35,-81,32 | 10 | 8 | 2 | 19:-30,-81,27 |

For each of the three groups of post-hocs (CTRL-MCS, CTRL-VS/UWS, MCS-VS/UWS) on low-alpha and for each cortical structure, both the number of activated voxels (significantly higher in one group with respect to the other, p <0.05, total number and splitted per hemisphere) and the voxel with the most extreme t-value, are presented. Only cortical structures for which at least one of the three groups of post-hocs led to an activation of at least 50% of the total number of voxels are presented. The first column lists the names of cortical structures whereas the second column lists the total number of voxels related to each cortical structure. For each of the three post-hocs groups, the first column reports the number of activated voxels in each cortical structure, the second column the number of activated voxels in the left hemisphere, the third column the number of activated voxels in the right hemisphere and the fourth column both the Brodmann area of the voxel with the most significant t-value and its Talairach coordinates (X,Y,Z).

**Table S2**

|  | **CTRL-MCS** | | | | **CTRL-VS/UWS** | | | | **MCS-VS/UWS** | | | |
| --- | --- | --- | --- | --- | --- | --- | --- | --- | --- | --- | --- | --- |
| **Cortical**  **structure** | **activated voxels** | | | **voxel with**  **higher t** | **activated voxels** | | | **voxel with**  **higher t** | **activated voxels** | | | **voxel with**  **higher t** |
|  | **Total** | **LH** | **RH** |  | **total** | **LH** | **RH** |  | **total** | **LH** | **RH** |  |
| **Postcentral Gyrus (363)** | 363 | 179 | 184 | 3:30,-22,43 | 363 | 179 | 184 | 3:25,-32,48 | 162 | 136 | 26 | 5:-35,-46,58 |
| **Precuneus (355)** | 355 | 161 | 161 | 31:20,-42,34 | 355 | 161 | 161 | 7:15,-41,48 | 310 | 161 | 116 | 19:-20,-81,41 |
| **Precentral Gyrus (357)** | 347 | 167 | 180 | 4:25,-27,47 | 317 | 160 | 157 | 4:25,-27,47 | 32 | 32 | 0 | 4:-30,-27,47 |
| **Middle Temporal Gyrus (359)** | 307 | 129 | 178 | 39:40,-57,21 | 275 | 145 | 130 | 39:35,-62,26 | 40 | 40 | 0 | 39:-35,-72,27 |
| **Superior Temporal Gyrus (419)** | 292 | 132 | 160 | 41:35,-33,15 | 238 | 126 | 112 | 39:35,-57,30 | 28 | 28 | 0 | 39:-35,-57,30 |
| **Cingulate Gyrus (287)** | 287 | 115 | 135 | 31:20,-22,38 | 268 | 106 | 127 | 31:5,-37,43 | 60 | 34 | 15 | 31:-15,-42,34 |
| **Inferior Parietal Lobule (286)** | 286 | 142 | 144 | 40:40,-32,43 | 286 | 142 | 144 | 40:35,-41,53 | 160 | 129 | 31 | 40:-35,-51,58 |
| **Middle Frontal Gyrus (494)** | 282 | 124 | 158 | 6:25,-8,42 | 182 | 85 | 97 | 6:-20,-12,60 | 0 | 0 | 0 | -- |
| **Cuneus (273)** | 273 | 124 | 124 | 7:10,-66,31 | 273 | 124 | 124 | 7:10,-66,31 | 223 | 124 | 74 | 19:-25,-86,37 |
| **Fusiform Gyrus (231)** | 230 | 114 | 116 | 37:35,-39,-6 | 224 | 115 | 109 | 19:25,-54,-10 | 22 | 22 | 0 | 18:-20,-88,-12 |
| **Superior Frontal Gyrus (368)** | 217 | 87 | 123 | 6:-10,-6,65 | 114 | 55 | 55 | 6:-20,-11,65 | 0 | 0 | 0 | -- |
| **Medial Frontal Gyrus (354)** | 217 | 82 | 111 | 6:15,-12,47 | 113 | 49 | 52 | 6:-10,-27,52 | 0 | 0 | 0 | -- |
| **Parahippocampal Gyrus (185)** | 185 | 93 | 92 | 27:15,-34,2 | 182 | 93 | 89 | 27:-10,-34,2 | 30 | 30 | 0 | 30:-25,-53,3 |
| **Insula (209)** | 182 | 86 | 96 | 13:30,-28,20 | 148 | 81 | 67 | 13:-30,-28,20 | 21 | 21 | 0 | 13:-30,-38,20 |
| **Lingual Gyrus (180)** | 180 | 84 | 87 | 18:20,-53,7 | 180 | 84 | 87 | 18:20,-53,7 | 111 | 83 | 19 | 17:-15,-82,4 |
| **Inferior Temporal Gyrus (158)** | 153 | 73 | 80 | 19:50,-58,-1 | 127 | 78 | 49 | 37:45,-68,-1 | 0 | 0 | 0 | -- |
| **Superior Parietal Lobule (134)** | 134 | 68 | 66 | 5:20,-41,57 | 134 | 68 | 66 | 5:20,-41,57 | 121 | 68 | 53 | 7:-25,-75,45 |
| **Middle Occipital Gyrus (145)** | 109 | 39 | 70 | 37:40,-63,3 | 145 | 75 | 70 | 19:30,-77,22 | 67 | 64 | 3 | 18:-15,-87,18 |
| **Posterior Cingulate (87)** | 87 | 39 | 40 | 23:5,-28,24 | 87 | 39 | 40 | 23:-5,-28,24 | 61 | 39 | 14 | 31:-10,-67,17 |
| **Paracentral Lobule (87)** | 87 | 33 | 34 | 31:10,-12,47 | 87 | 33 | 34 | 5:20,-41,48 | 51 | 25 | 17 | 5:-20,-41,48 |
| **Inferior Frontal Gyrus (367)** | 84 | 31 | 53 | 6:45,2,32 | 37 | 15 | 22 | 6:45,2,32 | 0 | 0 | 0 | -- |
| **Anterior Cingulate (141)** | 71 | 22 | 34 | 33:5,11,22 | 12 | 3 | 3 | 25:0,0,-4 | 0 | 0 | 0 | -- |
| **Uncus (61)** | 59 | 31 | 28 | 20:30,-16,-24 | 45 | 31 | 14 | 20:-30,-16,-29 | 0 | 0 | 0 | -- |
| **Sub-Gyral (57)** | 56 | 32 | 24 | 2:35,-27,38 | 51 | 30 | 21 | 40:25,-41,53 | 16 | 12 | 4 | 7:-25,-46,53 |
| **Supramarginal Gyrus (55)** | 55 | 28 | 27 | 40:40,-42,34 | 55 | 28 | 27 | 40:40,-42,34 | 28 | 28 | 0 | 40:-40,-42,34 |
| **Transverse Temporal Gyrus (36)** | 36 | 18 | 18 | 41:40,-33,15 | 36 | 18 | 18 | 41:40,-33,15 | 1 | 1 | 0 | 41:-35,-33,11 |
| **Inferior Occipital Gyrus (32)** | 28 | 13 | 15 | 19:40,-73,-5 | 32 | 17 | 15 | 19:40,-73,-5 | 17 | 17 | 0 | 17:-20,-93,-8 |
| **Angular Gyrus (27)** | 26 | 15 | 11 | 39:35,-61,35 | 27 | 16 | 11 | 39:35,-61,35 | 16 | 16 | 0 | 39:-30,-61,35 |
| **Subcallosal Gyrus (27)** | 20 | 9 | 9 | 25:5,4,-13 | 16 | 9 | 5 | 34:-10,4,-13 | 0 | 0 | 0 | -- |
| **Superior Occipital Gyrus (14)** | 11 | 5 | 6 | 19:35,-81,32 | 14 | 8 | 6 | 19:35,-81,32 | 8 | 8 | 0 | 19:-35,-81,32 |

For each of the three post-hoc groups of tests (CTRL-MCS, CTRL-VS/UWS, MCS-VS/UWS) on high-alpha and for each cortical structure, both the number of activated voxels (significantly higher in one group with respect to the other, p <0.05) and the voxel with the most extreme t-value, are presented. Only cortical structures for which at least one of the three post-hoc groups of tests led to an activation of at least 50% of the total number of voxels are presented. The first column lists the names of cortical structures whereas the second column lists the total number of voxels related to each cortical structure. For each of the three post-hoc groups, the first column reports the number of activated voxels in each cortical structure, the second column the number of activated voxels in the left hemisphere, the third column the number of activated voxels in the right hemisphere and the fourth column both the Brodmann area of the voxel with the most significant t-value and its Talairach coordinates (X,Y,Z).

**Phase coherence estimation**

Phase coherences between couples of electrodes were estimated for each trial in a 500ms window (50 to 550ms after the ocular blink) encompassing the BRO peak for the three bands of interest. As a first step, signals were filtered in the three bands with the same wavelet approach used in Bonfiglio et al., 2009. For each filtered signal, the instantaneous phase signal was estimated applying the Hilbert transform. Phase coherence between each pair of electrodes was computed following Rosenblum’s equation for coupled chaotic oscillators (see Rosenblum et al, 2004):

$$C_{ph}=\left(\frac{1}{N}\sum_{i=1}^{N} \sin\phi_{i}\right)^{2}+\left(\frac{1}{N}\sum_{i=1}^{N} \cos\phi_{i}\right)^{2}$$

where *N* is the number of samples and $\phi_{i}$ is the instantaneous phase difference between the signals at the two electrodes, estimated at time i. For each couple of electrodes and each band, the series of phase coherences values was submitted to a weighted ANOVA with GROUP (CTRL, MCS, VS/UWS) as a between-factor. P-values of each couple were estimated on the basis of 5000 permutations (Manly, 1997). Post-hocs were conducted for couples yielding a significant group-effect (unpaired t-tests with Sidak correction for multiple comparisons)

**References**

1. Bonfiglio L, Sello S, Andre P, Carboncini MC, Arrighi P, Rossi B (2009) Blink-related delta oscillations in the resting-state EEG: a wavelet analysis. Neurosci Lett 449:57-60

2. Rosenblum MG, Pikovsky AS, Kurths J (2004) Synchronization approach to analysis of biological systems. Fluctuation and Noise Letters 4(1):53-62

3. Manly BJF (1997) Randomization, bootstrap and Monte Carlo methods in biology. Chapman & Hall, London

**
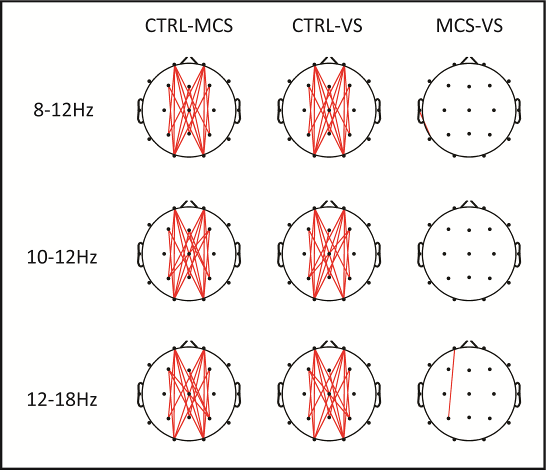
**

**Figure S6:** Results of post-hocs between phase coherences series of the three groups (CTRL, MCS, VS/UWS) are depicted for the three investigated bands. Red lines connect couples of electrodes yielding a significantly (p < 0.001) higher phase coherence in the former group than in the latter one (i.e. CTRL-MCS). Of all possible couples, only those consisting of a frontal/prefrontal electrode and of a posterior/occipital one are depicted.
