# Supplementary material for: Spectral Parameters Modulation and Source Localization of Blink-Related Alpha and Low-Beta Oscillations Differentiate Minimally Conscious State from Vegetative State/Unresponsive Wakefulness Syndrome
Source: PLoS One. 2014 Mar 27;9(3):e93252. doi: 10.1371/journal.pone.0093252 (PMC3970990; doi:10.1371/journal.pone.0093252)
Supplement: File S2 — Contains three exemplary EEG recordings in Matlab format, one related to a healthy subject, one to a MCS/UWS patient and one to a VS patient. (ZIP) [file pone.0093252.s002.zip › eeg files description.doc]

Each matlab file contains the raw trials of one subjects (the file name identifies the subject group). The file is structured as a matrix with each row corresponding to one EEG channel. For each channel, the series of three seconds trials are presented. The EEG is sampled at 256 Hz, each trials lasts 769 samples. Each matrix is composed by 20 rows (19 EEG channels and 1 EOG channel). A matlab localization file is included with labels and coordinates of the 19 EEG electrodes plus the EOG channel.
